# Task Prioritization in Dual-Tasking: Instructions versus Preferences

**DOI:** 10.1371/journal.pone.0158511

**Published:** 2016-07-08

**Authors:** Reinier J. Jansen, René van Egmond, Huib de Ridder

**Affiliations:** Faculty of Industrial Design Engineering, Delft University of Technology, Delft, The Netherlands; University of Groningen, NETHERLANDS

## Abstract

The role of task prioritization in performance tradeoffs during multi-tasking has received widespread attention. However, little is known on whether people have preferences regarding tasks, and if so, whether these preferences conflict with priority instructions. Three experiments were conducted with a high-speed driving game and an auditory memory task. In Experiment 1, participants did not receive priority instructions. Participants performed different sequences of single-task and dual-task conditions. Task performance was evaluated according to participants’ retrospective accounts on preferences. These preferences were reformulated as priority instructions in Experiments 2 and 3. The results showed that people differ in their preferences regarding task prioritization in an experimental setting, which can be overruled by priority instructions, but only after increased dual-task exposure. Additional measures of mental effort showed that performance tradeoffs had an impact on mental effort. The interpretation of these findings was used to explore an extension of Threaded Cognition Theory with Hockey’s Compensatory Control Model.

## Introduction

*“Police officer A*. *reflects on an incoming radio message*: *‘During an emergency call one receives a lot of information in a short timeframe*. *Such a call may include the shop name*, *crime type*, *potential dangers*, *suspect descriptions*, *which colleagues are on the case*, *and the plan*. *Meanwhile*, *you have to pay attention to the road*, *so sometimes you do not hear everything*.*’ His colleague comments on the imposed organizational demands*: *‘In case of solo patrol you have to be much sharper [*…*] but you will commit so many traffic violations*.*’”*

(field notes in [1])

This example illustrates a common situation in our daily lives, namely, that we are asked to perform several tasks at the same time. This multi-tasking, however, often requires too much attention resulting in a conflict referred to as task interference [2–5]. The obvious way to cope with task interference is to prioritize one task over the others [6]. But, as the police officers in the example show, this allocation of attention to one task goes at the expense of other tasks [7,8]. A possible solution was recently suggested by Salvucci & Taatgen in the form of continuous rapid switching between concurrent tasks [9]. Over time this will yield the impression that these tasks are performed simultaneously and hence reported as multi-tasking.

In this paper, we claim that the concept of rapid switching between concurrent tasks needs an extension in order to accommodate another aspect of the example with the police officers, namely, that they seem to have different preferences in task prioritization. The first police officer missed incoming radio messages because he preferred to prioritize the driving task while the other police officer committed traffic violations as a result of paying more attention to the radio messages. This suggests that people have internal preferences regarding task prioritization. The role of preference on task prioritization has received limited attention. It is typically assumed that task prioritization can be obtained by means of an external priority instruction on the relative importance of each task [4,10]. However, people are not always able or willing to follow priority instructions [11–14]. Cnossen et al. argue that judgments on performance decrements should be based on how people decide to prioritize between tasks, instead of what they are instructed to do [15]. In order to understand what really happened in these studies, we first need to know whether preferences do exist and whether they may have an impact on the effectivity of task priority instructions. The question thus becomes: is there a possibility that when people are instructed to prioritize one task over another, but in fact prefer to perform the other task, they act according to their preference?

The aim of the present study is to provide an answer to these questions by performing a series of experiments in which participants had to perform two concurrent tasks. The first step is to verify whether people have preferences (Experiment 1). The second step is to focus on possible interactions between preferences and instructions (Experiments 2 and 3). The findings of this quasi-experimental study called for a theoretical exploration. Therefore, as a third step, we extended Salvucci & Taatgen’s Threaded Cognition Theory [9] with Hockey’s Compensatory Control Theory [16,17] as a representation of cognitive-energetic models on task performance. But first, we introduce the mechanisms of task interference and task prioritization as predicted by Threaded Cognition Theory.

### Mechanism of task interference

Two tasks are said to interfere when simultaneous task execution results in decreased performance on one or both tasks (e.g., a tradeoff between missed radio items and traffic violations). Task interference is a convenient construct to investigate preferences in task prioritization, because task interference necessitates the process of task prioritization.

**Fig 1:**
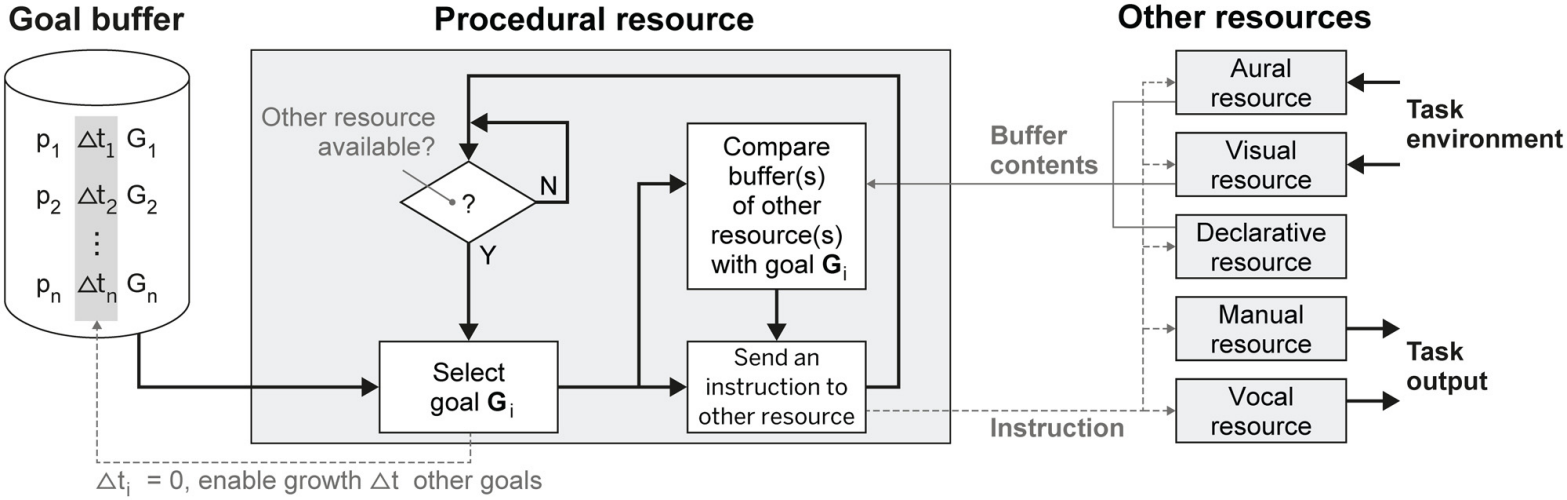
Control flowchart interpretation of Threaded Cognition Theory [9], with goal-related instructions fired by the procedural resource.

Threaded Cognition Theory (TCT) describes multi-tasking in terms of rapid switching (typically < 1 sec) between task goals in multiple resources [9]. Fig 1 presents three main components of TCT: the goal buffer, the procedural resource, and a set of five other resources. The goal buffer holds information about the current goals of the system. Each goal ‘G’ is associated with a priority level ‘p’ (expressed in percentages) and an idle time ‘Δt’. The procedural resource selects a goal from the goal buffer when one or more other resources are available. Details on the influence of p and Δt on goal selection are described in the next paragraph. The procedural resource integrates available information from the buffers of the other resources, and initiates new goal-related behavior by sending instructions. These instructions include sampling information from the task environment (e.g., aural and visual resources), storing and retrieving information (e.g., declarative resource), and taking action in accordance with the active goal (e.g., manual and vocal resources).

TCT explains task interference through an integration of two dominant perspectives on human-information processing. In line with Wickens’ [18,19] Multiple Resource Theory (MRT), task interference can take place in any of the resources. The total amount of task interference depends on the degree the demands of two tasks sharing common resources. For example, a combination of two visual/manual tasks results in more task interference than a visual/manual task with an aural/vocal task. In line with Pashler’s [20] Response Selection Bottleneck Theory (RSBT), each resource can only be used by one goal at a time. For example, the procedural resource sends an instruction to only one of the other resources at a time, and each procedural instruction requires approximately 50 ms of processing [21]. The serial processing that results from this bottleneck causes delays when two tasks have to be performed simultaneously.

### Goal selection in a dual-task situation

TCT literature provides two rules on goal selection by the procedural resource. First, when goals with an equal priority level simultaneously compete for the procedural resource, the least recently processed goal (i.e., with the lowest idle time Δt) claims right of way [9]. Second, when goals have unequal priority levels, the goal with the highest priority p claims the procedural resource, whereas alternative goals have to wait until the procedural resource is available again [21]. Furthermore, Salvucci & Beltowska [21] suggest that a generalized view on resource scheduling can be obtained by extending the priority level from a binary variable (e.g., high vs. low) to a continuous variable. The question then becomes which of the above two rules ‘wins’, when multiple goals have priority levels greater than zero.

Two additional mechanisms may influence goal selection. The Memory for Goals theory [22] relates prolonged goal inactivity (i.e., several seconds) with decay in goal activation, resulting in a decreased chance of goal selection. However, we do not expect prolonged goal inactivity in concurrent dual-tasking, because goals are likely to be reselected within a few seconds [23]. As a second mechanism, internal cues (e.g., cognitive chunking of phone numbers) and external cues (e.g., visual flow while driving) strengthen goal activation in memory (e.g., dialing, driving) [22,24]. We do acknowledge that cues may influence task prioritization, but if we position these effects in the goal buffer of Fig 1, then describing goal selection by TCT can be confined to aforementioned rules on p and Δt.

**Fig 2:**
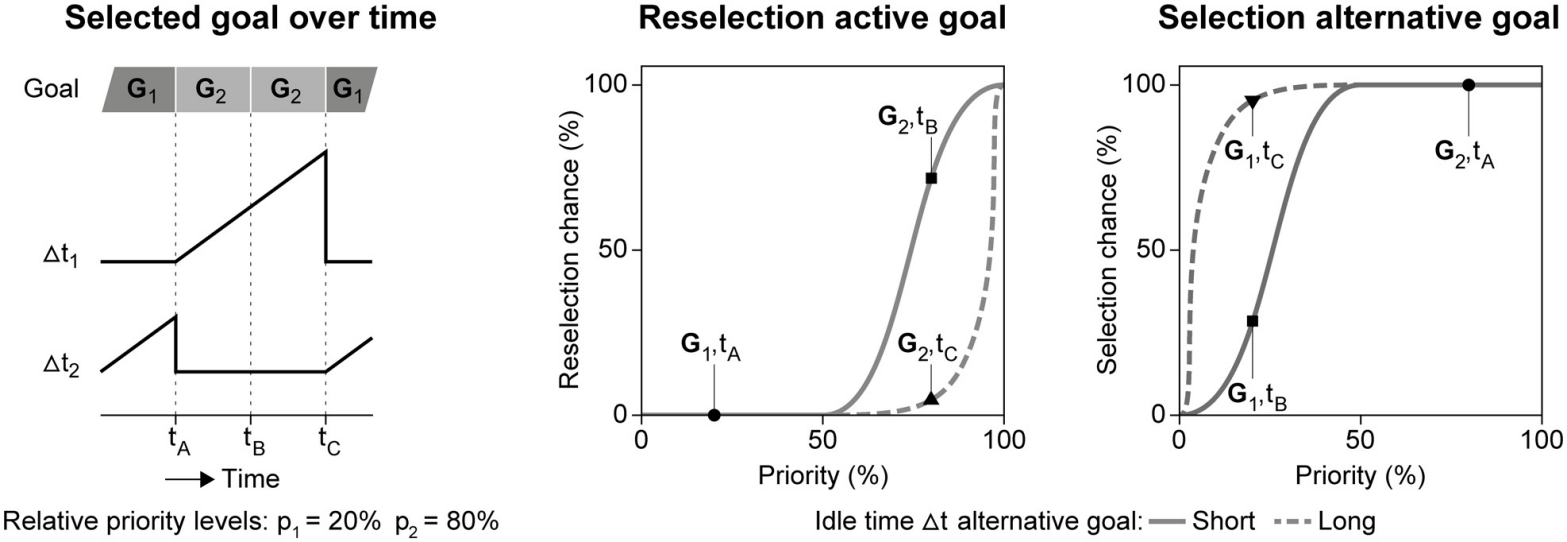
Goal selection chance. Goal selection by the procedural resource in a concurrent dual-task setting as function of goal priority p and idle time Δt since the goal was last selected.

We interpret goal selection in concurrent dual-tasking in terms of a chance mechanism. The reselection chance of an active goal G depends on its priority level p, and decreases with the idle time Δt of alternative goals. Fig 2 describes a dual-task scenario to illustrate the tradeoff between p and Δt. In this scenario the priority levels of each goal are fixed (i.c., p_1_ = 20%, p_2_ = 80%), whereas idle time per goal changes as function of time (see left panel). The idle time of an active goal is kept at zero (i.e., it is no longer idle when selected), whereas it increases autonomously for alternative goals (see Fig 1, left dashed arrow). The values of p and Δt are evaluated when the procedural resource has finished sending an instruction (e.g., at timestamps t_A_, t_B_, t_C_ in Fig 2). In our example, G_1_ is initially the active goal. The chance of reselection at t_A_ equals zero, because p_1_ is relatively low, and Δt_2_ > Δt_1_. Hence, G_2_ becomes the active goal. At t_B_ the active goal G_2_ is reselected, even though Δt_1_ > Δt_2_. The reason is that p_2_ is relatively high. However, at t_c_ the S-curves have shifted in favor of the alternative goal (see dashed lines in Fig 2), because Δt_1_ increased even further (i.e., Δt_1_ >> Δt_2_). Priority level p_2_ remains relatively high, but the reselection chance drops from point ‘G_2_,t_B_’ to ‘G_2_,t_C_’. Consequently, alternative goal G_1_ has a higher selection chance, despite its low priority. This scenario illustrates how a high priority goal is reselected several times, but eventually it loses out against a low priority goal, to be selected again soon. Moreover, the scenario shows how goal priority levels can be interpreted as continuous variables within TCT, which means a preference to prioritize one task over another task does not exclude briefly attending the lower-priority task.

### Paradigm

The experimental tasks in our study have been designed to ensure task interference, and consequently, task prioritization. Two continuous tasks have been used, based on observations in the context of police work [1]: a high speed driving task and an auditory memory task. The self-paced driving task represented police emergency driving. Participants have been given a printed map with several destinations. They had to read the map to navigate to as many destinations as possible within a fixed amount of time. The experimenter-paced memory task represented the demands of attending dispatcher-controlled police radio messages. Participants had to answer questions related to radio news items.

According to TCT, and based on empirical findings [21], task interference is expected to occur in shared resources. In our study, the driving task requires the visual resource (i.c., attention and processing), manual resource (i.c., motor control of the hands), declarative resource (i.c., to remember the current destination), and the procedural resource (i.c., sending instructions to the other resources). In addition, map reading requires mental rotation [25], which also places demands on the procedural resource. The memory task requires the auditory resource (i.c., attention and processing), verbal resource (i.c., to respond), declarative resource (i.c., to memorize chunks of information), and the procedural resource (i.c., to compare a memory question with the memorized chunks of information). In sum, task interference is expected in the procedural resource and the declarative resource.

Relative priority levels across the task goals determine which task suffers most from task interference (see p_1_ and p_2_ in Fig 2). However, TCT does not describe how these priority levels are set. One solution is to view task prioritization as a process on a strategic level (i.e., at a lower temporal resolution than TCT). Part of the driving skill is to strategically pay attention to other tasks for limited durations of time. Drivers can adapt the speed of their vehicle with an immediate effect on the difficulty of the (self-paced) driving task [26]. Alternatively, they can choose to ignore the (externally paced) secondary task [27]. Our experimental setup enables such strategic leverage to control the relative priority levels between the driving task and the memory task.

In the context of police work, performance differences resulting from preferences on task prioritization should be examined over periods of time comparable with the duration of an emergency response (i.e., minutes). In this context, the millisecond time window of TCT may seem out of place. However, Salvucci & Taatgen [9] demonstrate that TCT successfully predicts the consequences of task interference in continuous tasks, by extrapolating relatively short delays (<1 sec.) to aggregate performance measures (>>1 sec). In the present study, the presence of task interference is established by comparing aggregate performance measures of dual-task conditions with single-task conditions [10]. Task preferences should be reflected in distinct tradeoffs between the proportion of destinations reached, and the proportion of correct answers. Likewise, priority instructions should result in different tradeoffs. The effectivity of preferences and priority instructions has been analyzed through the corresponding interaction effects with task conditions (i.e., dual-task versus single-task).

Experiment 1 first investigates whether task interference occurs as pre-requisite for task prioritization. This is followed by an exploration on whether participants have preferences for tasks in absence of priority instructions, and whether preferences are reflected in task performance tradeoffs. Experiment 2 replicates Experiment 1, except that the former preferences are reformulated as priority instructions, and mental effort is taken into account. Experiment 3 tests two hypotheses on why the priority instructions in Experiment 2 did not yield significant results. Finally, the findings were used to explore an integration of TCT and Hockey’s [16,17] Compensatory Control Model.

## Experiment 1

The goal of Experiment 1 was to examine whether people have preferences in task prioritization. No task priority instructions were given. Preferences were inquired afterwards. Differences in preferences were examined by comparing the relative impact of interference between the tasks.

### Method

#### Participants

Twenty-one students of the Faculty of Industrial Design Engineering volunteered (17 males, 4 females, 20 to 35 years old, average 26.1 years). This study was approved by the Ethical Committee of Delft University of Technology. Participants gave written informed consent. All were native Dutch speakers. They reported normal hearing, and normal or corrected-to-normal vision.

#### Auditory memory task

Twenty-seven auditory stimuli were prepared, of which three were used for training. They consisted of Dutch news items (average duration: 15.2 sec), recorded by professional newsreaders. For each news item, a factual question was recorded by a native speaker from the Netherlands. Questions were related to information items close to the center of the corresponding news item, and allowed one correct answer. For example, the item: *“In the third quarter of this year less cars were sold than in the same period of last year*. *To be precise*: *six percent less*. *The trade organizations also expect a decrease in sales next year*.*”* was followed by the question: *“How many percent less cars were sold*?*”*. The stimuli and questions were saved as wave files (16 bit, 44.1 kHz). The goal of the memory task was to answer a question for each stimulus.

#### Driving task

The ‘RC Mini Racers’ [28] game was used for the driving task. The game featured a miniature vehicle in a closed environment without moving objects. Arrow keys controlled the vehicle. A test map was created for navigation. Seventeen labelled destinations (A-Q) were added to this map, adjacent to landmarks in the driving environment (e.g., the corner of a parking lot, a billboard). In addition, a training map was created with three labelled alternative destinations. The goal of the driving task was to drive from the starting location to as many destinations as possible in alphabetical order. Each time a destination was reached, a button had to be pressed to return to the starting location. A pilot study revealed that with extensive practice, a maximum of fifteen destinations can be reached.

#### Apparatus

The driving game ran on an Apple MacBook Pro 15”, placed on a table in a well-lit, quiet room. The maps, printed on A3 size paper, were positioned next to the laptop. Screen activity was recorded to verify whether the car was at the correct location in each attempt. Driving sounds and auditory stimuli were played through a pair of Creative Gigaworks T20 Series II loudspeakers, positioned at ear height, and approximately 30 cm to the left and right of the laptop. The experiment was conducted using a dedicated Max program.

#### Measures

Auditory memory performance was calculated as the proportion of correct answers within each experimental condition. Driving performance per experimental condition was calculated as the proportion of destinations reached, where *n* = 15 corresponds with 100%. Only correct attempts were included to calculate driving performance. For example, if the vehicle was placed north of a billboard, whereas the destination on the map was south of that billboard, the attempt was evaluated as incorrect, and excluded from subsequent analysis. For statistical analysis, the proportions were transformed with an arcsine transformation [29]. All statistical tests were conducted with SPSS v.22, and results were compared to an α level of .05. Type III sums of squares were used in all ANOVAs to compensate for differences in sample size.

#### Experimental design

**Fig 3:**
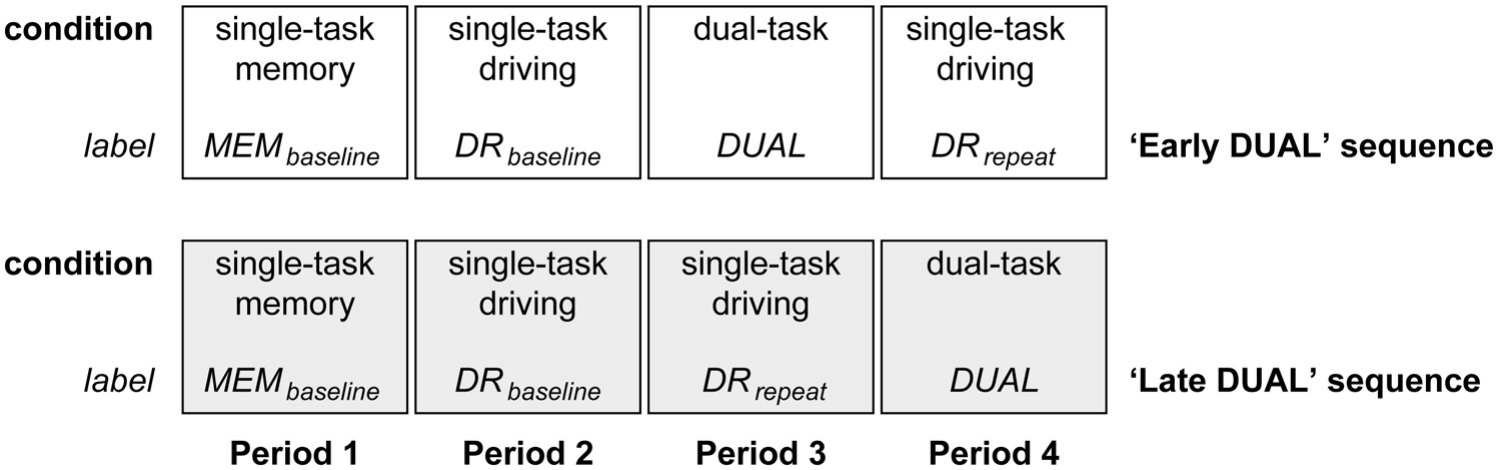
Experimental design.

The experiment consisted of two tasks: an auditory memory task, and a driving task. A crossover design was used with four periods, three experimental conditions, and two treatment sequences, see Fig 3.

The first two periods concerned single-task baseline performance on the memory task (i.e., condition MEM_baseline_), and on the driving task (i.e., condition DR_baseline_). The remaining two periods were ordered in two sequences to discriminate between dual task effects and potential learning effects on the driving task. In the ‘early DUAL’ sequence, the third period was a dual-task condition (i.e., condition DUAL), and the fourth period was a repetition of the single-task driving condition (i.e., labelled as DR_repeat_). This order was reversed in the ‘late DUAL’ sequence (i.e., DR_repeat_ followed by DUAL). Participants were randomly distributed over the ‘early DUAL’ (*n* = 11) and the ‘late DUAL’ (*n* = 10) sequences. Driving task performance was analyzed by comparing Period 2, 3, and 4. Memory performance was analyzed by comparing Period 1 with the dual-task conditions in Periods 3 and 4.

#### Procedure

The duration of the experimental conditions (i.e., MEM_baseline_, DR_baseline_, DR_repeat_, DUAL) was 5 minutes each. The auditory memory task ran automatically, and the driving task was self-paced. A beep sound was played to denote the end of an experimental condition.

After signing informed consent, a participant rehearsed the memory task for two minutes with three training stimuli. Memory questions were followed by a 4.5 sec answer time, a beep sound, and a 1.2 sec silence. Volumes across news items and questions were matched, and set to a comfortable listening level. A participant was instructed to verbalize an answer after each question. Responding after the beep sound was allowed if needed, but it was recommended to prepare for the next stimulus. In the MEM_baseline_ condition, 12 stimuli were randomly selected per participant from 24 test stimuli, and presented in random order.

Familiarization with the driving task lasted approximately ten minutes. First, the participant drove five laps in a racing game mode to get used to the controls. Next, the navigation subtask was rehearsed on the training map, with specific attention to correct and incorrect attempts. Game sounds were included for feedback on driving speed, but their volume was set to a low level to ensure audibility of the auditory stimuli in the upcoming DUAL condition.

In the DR_baseline_ condition and in subsequent conditions the training map was replaced with the test map. The execution order of the DR_repeat_ and DUAL conditions depended on the allocated sequence. In the DUAL condition, the remaining 12 stimuli of the memory task were presented in random order. No task priority instructions were given. At the end of the session, a participant was asked to which task attention was mostly paid in the DUAL condition (i.e., driving task, memory task, or both), and how this allocation policy was executed.

### Results

The presence of task interference was checked to ensure the necessity of task prioritization. Verbal reports on attention revealed two preferences regarding task prioritization. Finally, it was examined whether preferences are reflected in performance tradeoffs.

#### Task interference

Task interference is established when performance of one task is hindered by the addition of another task. Table 1 summarizes the results of a 2 (Sequence) × 2 (Period) mixed ANOVA on memory performance, and of a 2 (Sequence) × 3 (Period) mixed ANOVA on driving performance.

**Table 1:** Summary of ANOVA results on performance as function of sequence.

	Memory performance			Driving performance		
Source	*F*(1,19)	*p*	*η*_p_^2^	*F*(2,38)	*p*	*η*_p_^2^
Period	10.57	.004	.36	13.30	< .001	.41
Sequence	.081	.78	.004	.49	.49	.025
Per × Seq	.84	.37	.042	10.52	< .001	.36

Per = Period, Seq = Sequence.

**Fig 4:**
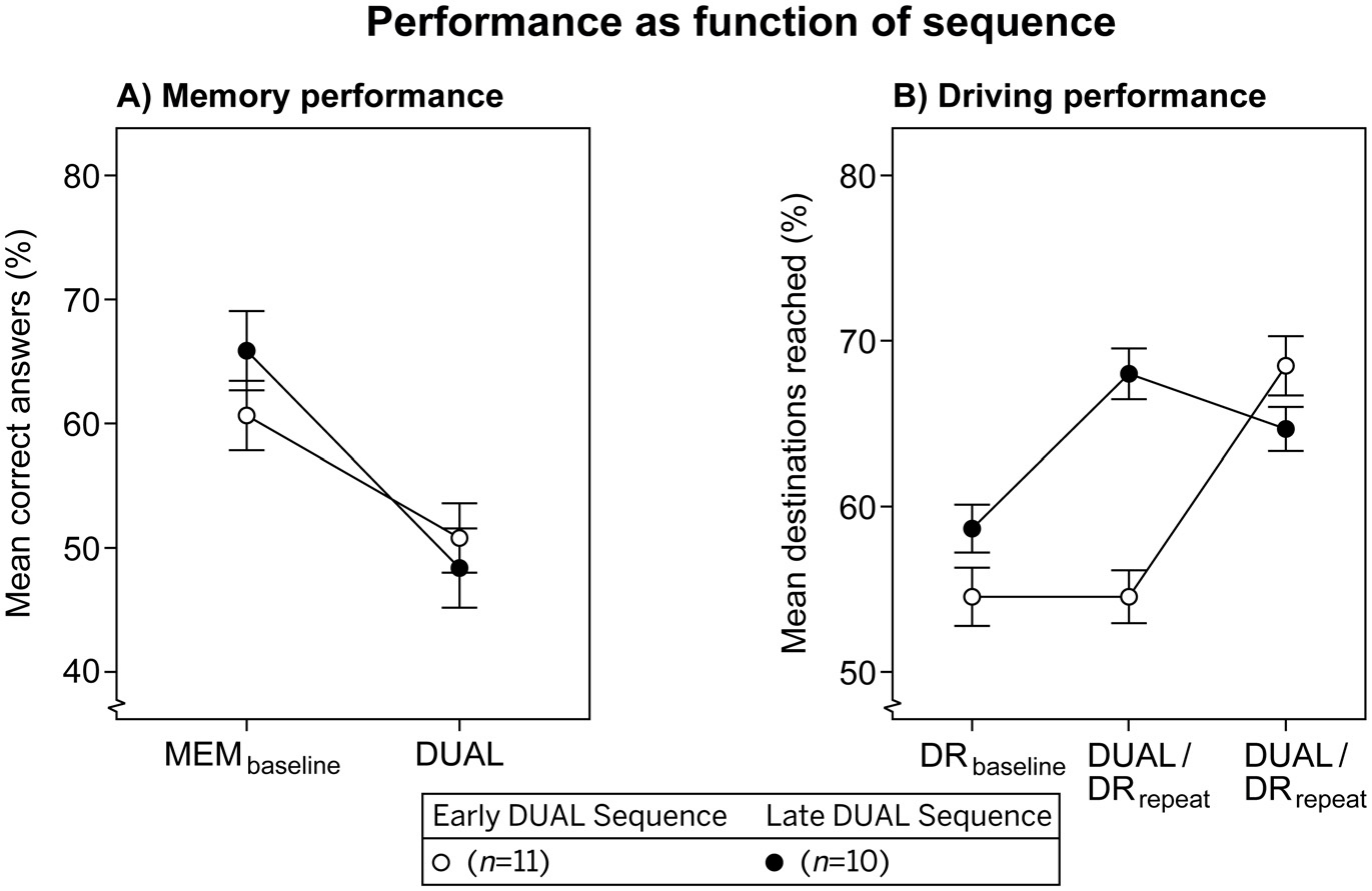
Memory task performance (A) and driving task performance (B) as function of sequence. Lines are added for interpretation only. Error bars represent +/- 1 standard error of the mean, corrected for within-subjects variability. Note: participants did not receive priority instructions.

Memory performance in the MEM_baseline_ condition (i.e., Period 1) was 63.10% (*SE* = 3.80), see Fig 4A. This value indicates that the memory task was a difficult one. Memory performance dropped significantly in the DUAL condition (*M* = 49.60%, *SE* = 3.17), which implies task interference.

The car was nearby the labelled destination in 96.8% of the attempts. Only these attempts were analyzed. The maximum number of correct attempts within a period was 14, and this number was attained by one participant only. Fig 4B shows that driving performance increases over time. A significant main effect of Period was found. Repeated type contrasts revealed that performance increased significantly from Period 2 (*M* = 56.51%, *SE* = 3.14) to Period 3 (*M* = 60.95%, *SE* = 3.45), *F*(1,19) = 5.34, *p* = .032, *η*_p_^2^ = .22, as well as from Period 3 to Period 4 (*M* = 66.67%, *SE* = 3.44), *F*(1,19) = 7.74, *p* < .012, *η*_p_^2^ = .30. This finding suggests an overall learning curve on the driving task.

A significant Period × Sequence interaction demonstrates that this learning process on the driving task was negatively influenced by the presence of the auditory memory task. For this there are two indications. First, from Period 2 to 3, participants in the ‘early DUAL’ sequence show stable performance from DR_baseline_ to DUAL, whereas the ‘late DUAL’ sequence shows improved performance from DR_baseline_ to DR_repeat_, *F*(1,19) = 6.24, *p* = .022, *η*_p_^2^ = .25. Second, from Period 3 to 4, the ‘early DUAL’ sequence shows improved performance from DUAL to DR_repeat_, whereas the ‘late DUAL’ sequence does not from DR_repeat_ to DUAL, *F*(1,19) = 19.81, *p* < .001, *η*_p_^2^ = .51. To summarize, the experimental setup resulted in bi-directional task interference. Memory performance was reduced by the addition of the driving task, whereas driving performance was hindered by the addition of the memory task.

#### Verbal reports on preference

Two types of verbal reports on the allocation of attention were found. Thirteen participants indicated that they paid most attention to the driving task, because they considered the driving task more rewarding, and the auditory memory task less important, and distracting. Furthermore, these participants viewed driving as an active task that could not be aborted, whereas the memory task could be ignored. We interpret these reports as a preference for the driving task (hereafter, ‘driving’ preference). Eight participants reported that they were motivated to perform both tasks as good as possible, and how they continuously switched attention between the tasks. We interpret these reports as an ‘equal’ preference for both tasks. In the ‘early DUAL’ sequence, the ‘driving’ and ‘equal’ preferences were found for seven and four participants, respectively. In the ‘late DUAL’ sequence, six participants had a ‘driving’ preference, and four participants had an ‘equal’ preference. The preference distributions were not significantly different between the ‘early DUAL’ and ‘late DUAL’ sequences (*P* = 1.00, Fisher’s exact test).

#### Preferences versus tradeoffs

Now that two preferences regarding task prioritization have been found, the next question is whether these preferences are reflected in performance. Such reflection should be visible in the interaction between Preference and Period, because not all conditions required task prioritization. Table 2 summarizes the results of a 2×2×2 mixed ANOVA on memory performance, with Preference and Sequence as between-subjects factors, and Period as within-subjects factor. Table 2 also includes the results of a 2 (Preference) × 2 (Sequence) × 3 (Period) mixed ANOVA on driving performance. Task interference is once again demonstrated by a signifant effect of Period on memory performance, and by significant effects of Period and Period × Sequence on driving performance.

**Table 2:** Summary of ANOVA results on performance as function of sequence and preference.

	Memory performance			Driving performance		
Source	*F*(1,17)	*p*	*η*_p_^2^	*F*(2,34)	*p*	*η*_p_^2^
Period	9.45	.007	.36	12.36	< .001	.42
Sequence	.13	.72	.008	.26	.62	.015
Preference	1.88	.19	.099	.20	.66	.011
Per × Seq	1.29	.27	.07	11.47	< .001	.40
Per × Pref	11.32	.004	.40	5.64	.008	.25
Seq × Pref	.023	.88	.001	.48	.50	.027
Per × Seq × Pref	.097	.76	.006	.70	.51	.039

Per = Period, Pref = Preference, Seq = Sequence.

**Fig 5:**
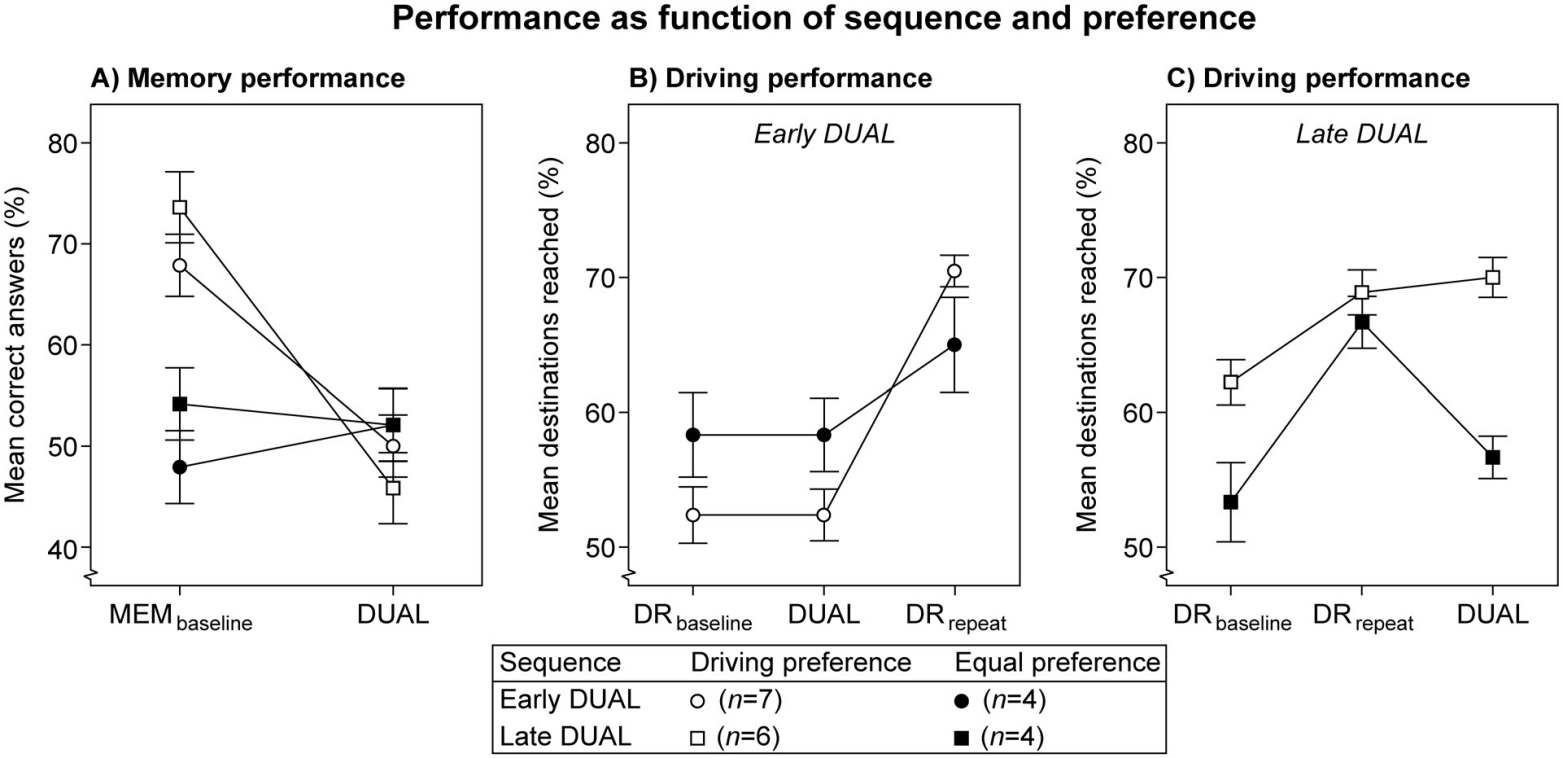
Memory task performance (A) and driving task performance (B,C) as function of sequence and preference. Lines are added for interpretation only. Error bars represent +/- 1 standard error of the mean, corrected for within-subjects variability. Note: participants did not receive priority instructions.

In Fig 5A the ‘equal’ preference (represented with filled circles and squares) shows stable memory performance from MEM_baseline_ to DUAL, whereas memory performance strongly decreases with the ‘driving’ preference (open circles and squares). This observation was confirmed by a significant Preference × Period interaction. In addition, participants with a ‘driving’ preference (*M* = 59.33%, *SE* = 3.68) appear to have a higher memory performance than those with an ‘equal’ preference (*M* = 51.56%, *SE* = 4.67), which is caused by differences in the MEM_baseline_ condition. A separate 2 (Preference) × 2 (Sequence) ANOVA on MEM_baseline_ data yielded a significant effect of Preference, *F*(1,17) = 8.16, *p* = .011, *η*_p_^2^ = .32. The other sources of variance were non-significant.

Fig 5B and 5C show the mean percentages of destinations reached for participants with an ‘early DUAL’ and a ‘late DUAL’ sequence, respectively. A significant interaction between Preference and Period was found. Repeated contrasts revealed that this interaction was only significant from Period 3 to Period 4, *F*(1,17) = 13.12, *p* = .002, *η*_p_^2^ = .44. Driving performance in the ‘late DUAL’ sequence shows an interaction between Preference and Period (see Fig 5C). Performance drops from DR_repeat_ to DUAL for the ‘equal’ preference (closed squares), but not for the ‘driving’ preference (open squares). This interaction seems absent for participants with an ‘early DUAL’ sequence (see Fig 5B).

In summary, the presence of task interference necessitated task prioritization. Significant interactions between Preference and Period were found on both memory performance and driving performance, which demonstrates that preferences resulted in different performance tradeoffs.

### Discussion

The two main findings of Experiment 1 are that participants have prioritization preferences in a situation of task interference (i.e., a ‘driving’ preference and an ‘equal’ preference), and that the inquired preferences are reflected in actual performance tradeoffs. The ‘driving’ preference inhibits task interference of the memory task on the driving task. However, this inhibition has only been found in the ‘late DUAL’ sequence, which suggests that increased exposure to the driving task is required for effective use of preferences.

The driving performance data strongly suggest a learning curve on the driving task, which has been accounted for by using two task sequences. Nonetheless, the learning curve may have been incomplete by the time participants performed the DR_repeat_ ('early DUAL' sequence) or DUAL ('late DUAL' sequence) condition. As a result, it is not possible to conclude whether the 'driving' preference fully, or only partially, mitigates the interference of the memory task on the driving task. Therefore, Experiment 2 incorporates a single-task control group to investigate the learning curve on the driving task in absence of the memory task.

Perceived task utility appears to be a recurring theme in the verbal reports that were used to inquire preferences. In the transition from single-task to dual-task driving, participants with an 'equal' preference may have considered the memory task an appealing alternative to the driving task, resulting in sustained memory performance at the cost of decreased driving performance (cf. [16,30]). However, it is not possible to conclude whether the ‘equal’ preference actually mitigates the interference of the driving task on the memory task, because of differences in baseline performance. One participant group may have had better memory performance skills. Another potential factor is that participants in one group have spent more effort on the task to compensate for the perceived task demands, in line with cognitive-energetic models on task performance [16,30,31,32,33]. Experiments 2 and 3 address effort-related adjustment by also including measurements of mental effort.

## Experiment 2

Experiment 1 revealed two preferences, which were reflected in performance tradeoffs at the late dual-task treatment sequence. The goal of Experiment 2 was to examine whether using these preferences as priority instructions results in similar performance tradeoffs. The 'late DUAL' sequence of Experiment 1 was used, because preferences were not manifested in driving performance in the 'early DUAL' sequence. A control group without any instructions was added to discriminate between dual task effects and learning effects, akin to the use of two task sequences in Experiment 1.

### Method

The driving task and measures were identical to Experiment 1.

#### Participants

Thirty-four students of the Faculty of Industrial Design Engineering volunteered (25 males, 9 females, 18 to 31 years old, average 23.4 years). This study was approved by the Ethical Committee of Delft University of Technology. Participants gave written informed consent. Participants were native Dutch speakers, and reported normal or corrected-to-normal vision. No hearing problems were reported.

#### Auditory memory task

The number of training stimuli was increased from three to twelve to reduce potential differences in baseline performance. Apart from that, the auditory memory task was identical to Experiment 1.

#### Apparatus

The Max program of Experiment 1 was extended with the subjective Rating Scale Mental Effort (RSME) [34]. This scale has a range from 0 to 150, and is accompanied by Dutch anchor words. A translated version can be found in S1 Fig.

#### Experimental design

Participants were randomly distributed over a ‘driving’ instruction (*n* = 12), an ‘equal’ instruction (*n* = 11), and a control group without an instruction (*n* = 11). The ‘late DUAL’ sequence of Experiment 1 was used for the 'driving' and 'equal' instruction groups: MEM_baseline_-DR_baseline_-DR_repeat_-DUAL_instr_. The control group did not include a dual-task condition, but instead it featured two additional single-task conditions: MEM_baseline_-DR_baseline_-DR_repeat_-MEM_repeat_-DR_repeat2_.

#### Procedure

Two modifications were made to the procedure of Experiment 1. A priority instruction was given before the DUAL_instr_ condition. Participants with the ‘driving’ instruction had to prioritize the driving task. They were invited to perform the memory task, but only if this would not degrade driving task performance. Participants with an ‘equal’ instruction had to treat both tasks as equally important by performing as good as possible on both tasks. Participants in the control group did not receive a priority instruction, because no dual-task condition was involved. Finally, subjective mental effort was administered after each condition with an onscreen RSME

### Results

Analogous to Experiment 1, the presence of task interference was checked to ensure the necessity of task prioritization. This was followed by an examination into the effect of the 'driving' and 'equal' instructions on tradeoffs between performance and mental effort. Finally, the control group of this experiment was compared with the data of Experiment 1 to investigate learning effects.

#### Task interference

**Fig 6:**
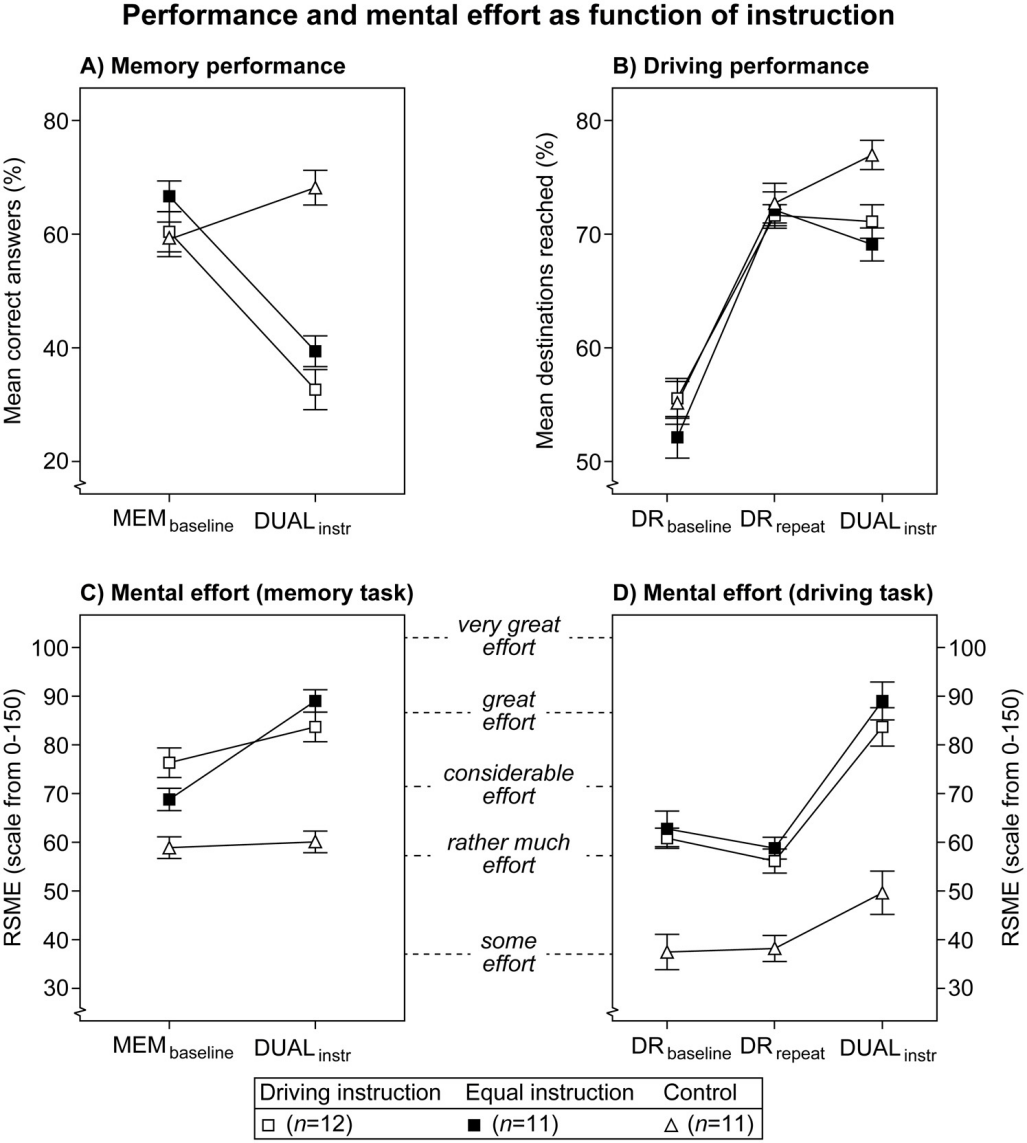
Memory task performance (A), driving task performance (B) and subjective mental effort (C,D) as function of instruction. Lines are added for interpretation only. Error bars represent +/- 1 standard error of the mean, corrected for within-subject variability.

Fig 6A and 6B show memory performance and driving performance, respectively. For driving performance, the maximum number of correct attempts within a period was 15, and only these correct attempts were analyzed. Fig 6C and 6D show mental effort related to the memory task and the driving task, respectively. Across these graphs the same mental effort data are used for the 'driving' and 'equal' instructions in the DUAL_instr_ condition. For the control group, however, the mental effort data of the single-task MEM_repeat_ and DR_repeat2_ conditions are used for comparisons in the DUAL_instr_ condition.

Fig 6A shows that, for the 'driving' and 'equal' instructions, memory performance clearly decreases from MEM_baseline_ to DUAL_instr_, whereas the control group shows stable performance. Driving performance in Fig 6B increases similarly from DR_baseline_ to DR_repeat_ for all groups, and then remains relatively stable from DR_repeat_ to DUAL_instr_, whereas the performance tends to increase for the control group. For the 'driving' and 'equal' instructions, these transitions come at the expense of increased mental effort from MEM_baseline_ (anchor word: 'considerable effort') to DUAL_instr_ (anchor word: ‘great effort’), and increased mental effort from DR_repeat_ (anchor word: ‘rather much effort’) to DUAL_instr_ (anchor word: 'great effort') (see Fig 6C and 6D). The control group, however, shows stable mental effort on the memory task, and relatively stable mental effort on the driving task. At both tasks the ratings of the control group appear to be lower than the other instruction groups, with substantial higher mental effort for the memory task than for the driving task. The observed tradeoff between memory performance and mental effort indicates dual-task interference between the memory task and the driving task.

These observations were supported by the results of a mixed 3 (Instruction) × 2 (Period) ANOVA on memory performance, and a mixed 3 (Instruction) × 3 (Period) ANOVA on driving performance. Both ANOVAs were also conducted on mental effort, corresponding with the memory task (i.e., 2 periods) and the driving task (i.e., 3 periods). Table 3 summarizes the results of these tests.

**Table 3:** Summary of ANOVA results on performance and mental effort as function of instruction.

	**Memory performance**				**Driving performance**			
**Source**	***df***	***F***	***p***	***η***_**p**_^**2**^	***df***	***F***	***p***	***η***_**p**_^**2**^
Period	(1,31)	17.28	< .001	.36	(2,62)	79.44	< .001	.72
Instruction	(2,31)	3.02	.064	.16	(4,62)	.31	.74	.019
Per × Instr	(2,31)	11.71	< .001	.43	(4,62)	1.89	.12	.11
	**Mental effort (memory task)**				**Mental effort (driving task)**			
**Source**	***df***	***F***	***p***	***η***_**p**_^**2**^	***df***	***F***	***p***	***η***_**p**_^**2**^
Period	(1,31)	3.56	.068	.10	(1.51, 46.77)	29.14	< .001	.49
Instruction	(2,31)	5.29	.011	.25	(2,31)	10.63	< .001	.41
Per × Instr	(2,31)	3.80	.033	.20	(3.02, 46.77)	1.65	.17	.096

Instr = Instruction, Per = Period. The *df* of mental effort on the driving task were adjusted using Greenhouse-Geisser, *ε* = .75.

Memory performance decreased significantly from MEM_baseline_ to DUAL_instr_, but a significant interaction between Period and Instruction shows that this was not the case for the control group. The above interaction was also significant on mental effort with the memory task. Fig 6C suggests that mental effort increases with the 'equal' instruction, whereas it remains stable in the control group.

Significant main effects of Period were found on driving performance, and on mental effort with the driving task. Repeated contrasts showed that driving performance increased significantly from DR_baseline_ to DR_repeat_, *F*(1,31) = 111.88, *p* < .001, *η*_p_^2^ = .78, but not from DR_repeat_ to DUAL_instr_, *n*.*s*. Mental effort, on the other hand, only increased significantly from DR_repeat_ to DUAL_instr_, *F*(1,31) = 44.51, *p* < .001, *η*_p_^2^ = .59. Fig 6D indicates that the 'driving' and 'equal' instruction groups were the main drivers for this effect. Thus, participants improved their driving performance without investing more mental effort, but mental effort increased when the memory task was added. The interaction between Period and Instruction on driving performance was non-significant, which indicates that the 'driving' and 'equal' instruction groups followed a similar learning curve as the control group.

In addition, two significant main effects of Instruction on mental effort were found. Fig 6C and 6D show that for both tasks the mental effort ratings of the control group are lower than the other instruction groups. Furthermore, Fig 6C and 6D suggest that mental effort was higher in the MEM_baseline_ condition than in the DR_baseline_ condition. This difference was confirmed through a two-way ANOVA with Instruction and Task as factors, which yielded a significant effect on Task, *F*(1,31) = 21.08, *p* < .001, *η*_p_^2^ = .41. This finding suggests that the memory task placed a heavier burden in the DUAL_instr_ condition than the driving task.

#### Instructions versus tradeoffs

Fig 6 shows a high degree of similarity on all measures between the ‘equal’ and ‘driving’ priority instructions. Although significant interactions between Instruction and Period were found, these were all related to differences with the control group. This also applies to the significant main effects of Instruction on mental effort. The absence of significant differences between the 'driving' and 'equal' instructions was not caused by differences between participant groups, as they performed similar in the single-task conditions, and showed similar mental effort ratings.

#### Comparison with learning curves from Experiment 1

The control group of Experiment 2 helps to understand the apparent learning curves in Experiment 1. Within the control group, a *t*-test did not reveal a significant difference in memory performance between MEM_baseline_ (*M* = 59.09%, *SE* = 4.42) and MEM_repeat_ (*M* = 68.18%, *SE* = 5.60). Furthermore, a repeated-measures one-way ANOVA showed a significant effect of Period on driving performance, *F*(2,20) = 26.67, p < .001, *η*_p_^2^ = .73. Driving performance increased significantly from DR_baseline_ (*M* = 55.15%, *SE* = 4.41) to DR_repeat_ (*M* = 72.73%, *SE* = 3.42), *F*(1,10) = 25.89, *p* < .001, *η*_p_^2^ = .72, but not from DR_repeat_ to DR_repeat2_ (*M* = 76.97%, *SE* = 4.25), *n*.*s*. These findings suggest that there is no learning curve on the memory task, whereas two experimental periods are required to fully learn the driving task.

**Fig 7:**
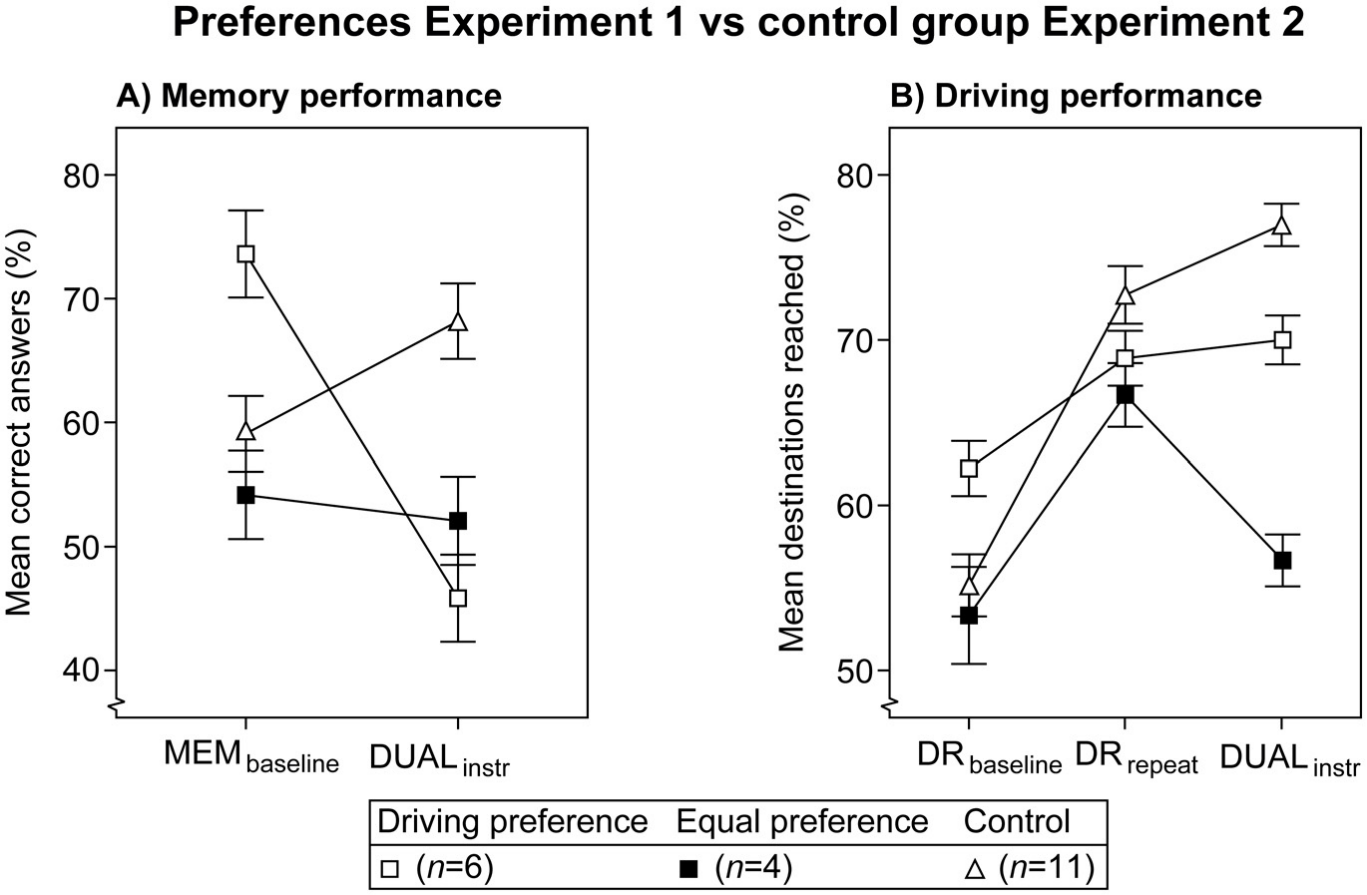
Performance by the control group (Experiment 2) versus two preferences (Experiment 1). Lines are added for interpretation only. Error bars represent +/- 1 standard error of the mean, corrected for within-subjects variability. Note: participants did not receive priority instructions.

In Fig 7 the control group is juxtaposed with the 'driving' and 'equal' preferences in the 'late DUAL' sequence of Experiment 1. Fig 7A shows that memory performance decreases with the 'driving' preference, whereas it remains relatively stable with the 'equal' preference and in the control group. All groups appear to have reached a similar driving performance level in the DR_repeat_ condition (see Fig 7B), which is consistent with the above statement on the driving task learning curve. Furthermore, driving performance decreases strongly with the 'equal' preference from DR_repeat_ to DUAL_instr_, whereas it remains stable with both the 'driving' preference and the control group.

The results of a 3×2 mixed ANOVA on memory performance and a 3×3 mixed ANOVA on driving performance support these observations (see Table 4). On memory performance a significant interaction between Preference and Period was found. In addition, a one-way ANOVA on the MEM_baseline_ condition did not reveal a significant difference in baseline performance between the preferences and the control group. This implies that participants with the 'equal' preference managed to protect memory performance as if no additional task was involved.

**Table 4:** Summary of ANOVA results on the control group (Experiment 2) versus two preferences (Experiment 1).

	Memory performance				Driving performance			
Source	*df*	*F*	*p*	*η*_p_^2^	*df*	*F*	*p*	*η*_p_^2^
Period	(1,18)	1.93	.18	.097	(2,36)	19.02	< .001	.51
Preference	(2,18)	.87	.44	.088	(2,18)	.94	.41	.095
Per × Pref	(2,18)	7.36	.005	.45	(4,36)	5.15	.002	.36

Per = Period, Pref = Preference.

The overall learning curve on driving performance was reflected in a significant main effect of Period. This effect was significant from DR_baseline_ to DR_repeat_, *F*(1,18) = 27.15, *p* < .001, *η*_p_^2^ = .60, but not from DR_repeat_ to DUAL_instr_, *n*.*s*. The interaction between Preference and Period was significant only from DR_repeat_ to DUAL_instr_, *F*(2,18) = 5.04, *p* = .018, *η*_p_^2^ = .36. A separate one-way ANOVA on the DR_repeat_ condition did not yield a significant effect, which means that the preference groups learned to perform the driving task at a similar level as the control group.

### Discussion

Experiment 2 has two main findings: a substantial increase in mental effort from single-task to dual-task conditions, and no effect of the manipulation of the 'driving' and 'equal' priority instructions. A comparison with the late dual-task sequence of Experiment 1 clarifies which instruction has not been followed. The ‘driving instruction’ shows a performance tradeoff similar to the ‘driving’ preference group: stabilized driving performance at the expense of decreased memory performance. A comparison with the control group confirms that decreased memory performance in both instruction groups could be attributed to dual-task interference.

Contrary to the 'driving' instruction group, the ‘equal’ instruction group shows a performance tradeoff dissimilar to its ‘equal’ preference counterpart. In fact, it resembles the performance tradeoff of the ‘driving’ preference group. Therefore, participants in Experiment 2 appear to have followed the ‘driving’ instruction, but not the ‘equal’ priority instruction. This is in line with the observed 3:2 distribution of the ‘driving’ and ‘equal’ preferences in Experiment 1, suggesting a majority of the participants in the ‘equal’ instruction group prefer the ‘driving’ instruction, and acting accordingly.

Next to these performance tradeoffs, it appears there has also been a tradeoff between performance and mental effort. In the transition from single-task to dual-task conditions, driving performance remains stable, but at the cost of decreased memory performance and increased mental effort. This tradeoff can be interpreted as a protection mechanism of the driving task against performance degradation. Such a protection mechanism has been described previously by the Compensatory Control Model [16,17], which predicts strategies involving secondary task decrements and increased mental effort. Interestingly, participants with a ‘driving’ preference in Experiment 1 reported the memory task as secondary to the driving task.

An additional factor that may explain why the ‘equal’ instruction was not followed is related to the experimental design. Potential effects resulting from the priority instructions may have been overshadowed by the increased demands associated with the single-task to dual-task transition. Support is found in a study by Liepelt et al. [35] on the effect of dual-task exposure on intertask coordination. The researchers let one participant group train two tasks separately (e.g., a visual/manual and an auditory/vocal task), whereas another group received a mixture of single-task and dual-task training conditions. Participants were instructed to prioritize both tasks equally. The latter group outperformed the former group on the auditory/vocal task in a dual-task test condition. Improved dual-task performance was related to accelerated task switching in the response selection stage (cf. RSBT), which could only be trained during dual-task conditions. These findings suggest that the ‘equal’ priority instruction in the present study may be effective after additional dual-task exposure, especially in relation to auditory memory performance.

## Experiment 3

The goal of Experiment 3 was to juxtapose preferences with priority instructions in the same experimental setup. Like the previous experiment, the manipulation of the priority instructions was evaluated through the interaction between Instruction and Period. However, this time two dual-task conditions were used: one condition without priority instructions, and one condition with. The possibility of conflicting preferences was taken into account by asking participants afterwards about their preference in the first dual-task condition.

In Experiment 2 we compared task performance with Experiment 1 to evaluate the resemblance between the priority instructions and the preferences on which the priority instructions were based. The addition of a second dual-task condition in Experiment 3 no longer allows for such a comparison with Experiment 1. Therefore, a ‘free choice’ group was added, that will not receive a priority instruction during the second dual-task condition.

### Method

The auditory memory task, driving task, apparatus, and measures, were identical to Experiment 2.

#### Participants

Forty-three students of the Faculty of Industrial Design Engineering volunteered for a €10,- reward (29 males, 14 females, 18 to 28 years old, average 21.3 years). This study was approved by the Ethical Committee of Delft University of Technology. Participants gave written informed consent. All were native Dutch speakers. They reported normal hearing, and normal or corrected-to-normal vision.

#### Experimental design

Participants were randomly distributed over three priority instructions: ‘driving’ (*n* = 14), ‘equal’ (*n* = 15), or ‘free choice’ (e.g., no instruction at all, *n* = 14). The following sequence was used: DR_baseline_-DUAL_baseline_-DUAL_instr_, in which DUAL_baseline_ concerned a dual-task baseline condition. The MEM_baseline_ condition was removed to ensure equal exposure across all Experiments. Such removal is legitimate, because the control group in Experiment 2 showed stable performance on the memory task.

#### Procedure

The procedure of Experiment 2 was modified. The memory task and the driving task were practiced as before (i.e., 12 training stimuli, a separate training map with 3 destinations). No priority instructions were given, except in the DUAL_instr_ condition. At the end of the session, the participant was asked to which task attention was mostly paid in the DUAL_baseline_ condition (i.e., driving task, memory task, or both tasks), and how this was executed.

### Results

One participant with the 'equal' instruction and one participant in the 'free choice' group were excluded from analysis, because they were unable to execute the tasks. First, we examined how the priority instructions were followed. Subsequent analyses investigated whether preferences influenced how these instructions were followed.

#### Instructions versus tradeoffs

**Fig 8:**
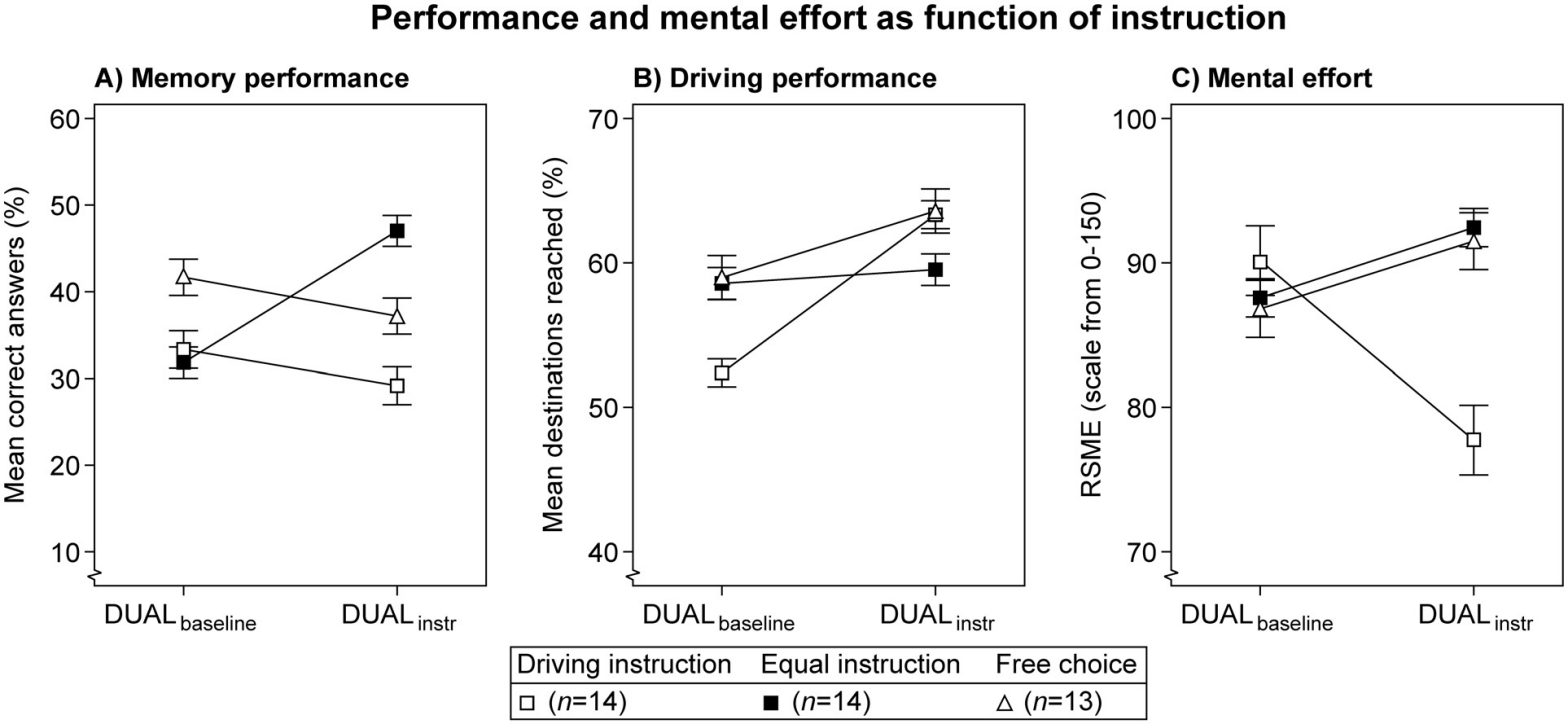
Performance and mental effort as function of instruction. Lines are added for interpretation only. Error bars represent +/- 1 standard error of the mean, corrected for within-subjects variability. Note: no instruction was provided in the DUAL_**baseline**_ condition.

A 3 (Instruction) × 2 (Period) mixed ANOVA was conducted to investigate the influence of Instruction on tradeoffs between performance and mental effort, see Table 5. Driving performance increased significantly from DUAL_baseline_ to DUAL_instr_, which is indicative for a learning effect (see Fig 8B). Furthermore, the interaction between Instruction and Period proved to be significant on all measures. Fig 8 shows that memory performance increases with the 'equal' instruction, at the cost of increased mental effort, and with stable driving performance. The 'driving' instruction, on the other hand, shows increased driving performance and decreased mental effort, at the cost of slightly decreasing memory performance. Finally, the 'free choice' group appears to mirror the 'driving' group on memory performance, but the 'equal' group on mental effort. The tradeoffs between memory performance and driving performance with the 'driving' and 'equal' instructions are in line with those found in Experiment 1, which means the instructions were followed as intended.

**Table 5:** Summary of ANOVA results on performance and mental effort as function of instruction.

		Memory performance			Driving performance			Mental effort		
Source	*df*	*F*	*p*	*η*_p_^2^	*F*	*p*	*η*_p_^2^	*F*	*p*	*η*_p_^2^
Period	(1,38)	.82	.37	.021	15.23	< .001	.29	.18	.68	.005
Preference	(2,38)	1.31	.28	.065	.18	.84	.009	.45	.64	.023
Per × Pref	(2,38)	7.02	.003	.27	3.93	.028	.17	6.52	.004	.26

Per = Period, Pref = Preference.

#### Verbal reports on preference

Although the instructions were apparently followed, participants may have differed in their preferences regarding task prioritization within each instruction group. The verbal reports of twenty-six participants on the DUAL_baseline_ condition were interpreted as ‘driving’ preference. These participants noted that the driving environment provided stronger cues than the news items in the background, that the implications of not paying attention to the driving task were more immediate, and that standing still was not an option. The driving task was also prioritized because it was considered easier and more interesting, whereas the news items were considered irrelevant during driving.

Fourteen verbal reports were interpreted as ‘equal’ preference. These participants reported a desire to combine the two tasks, and to avoid incorrect answers while reaching as many destinations as possible. Their approaches were described as driving slower to perform both tasks at the same time, and to frequently switch attention, but it was also noted that attending news items occasionally resulted in losing track on the driving task.

In addition, one participant in the ‘free choice’ group appeared to prefer the memory task. This participant showed results comparable to the ‘driving’ and ‘equal’ preference groups within the ‘free choice’ instruction, except that memory performance was relatively high (i.c., 67% at DUAL_baseline_, 79% at DUAL_instr_). Although a preference for the memory task apparently exists, its occurrence is rare (also see Experiments 1 and 2). Therefore, further analysis is restricted to the ‘driving’ and ‘equal’ preferences.

Table 6 shows the resulting distribution of preferences. No significant differences were found in the preference distributions between the instruction groups (*P* = .78, Fisher’s exact test). In addition, the preference distribution within the ‘free choice’ group was not significantly different from the preference distribution in Experiment 1 (*P* = 1.00, Fisher’s exact test). The next question, then, is whether these preferences influenced how the instructions were followed, just as they affected performance tradeoffs in Experiment 1.

**Table 6:** Participant distribution as function of task priority instruction and preference.

Priority instruction	Preference: driving	Preference: equal	Total
Driving	8	6	14
Equal	10	4	14
Free choice	8	4	12
Total	26	14	40

Note: Participants in the free choice group did not receive a task priority instruction. Not reported in this table is one participant in the free choice group, who preferred to prioritize the memory task.

#### Instructions versus preferences

**Fig 9:**
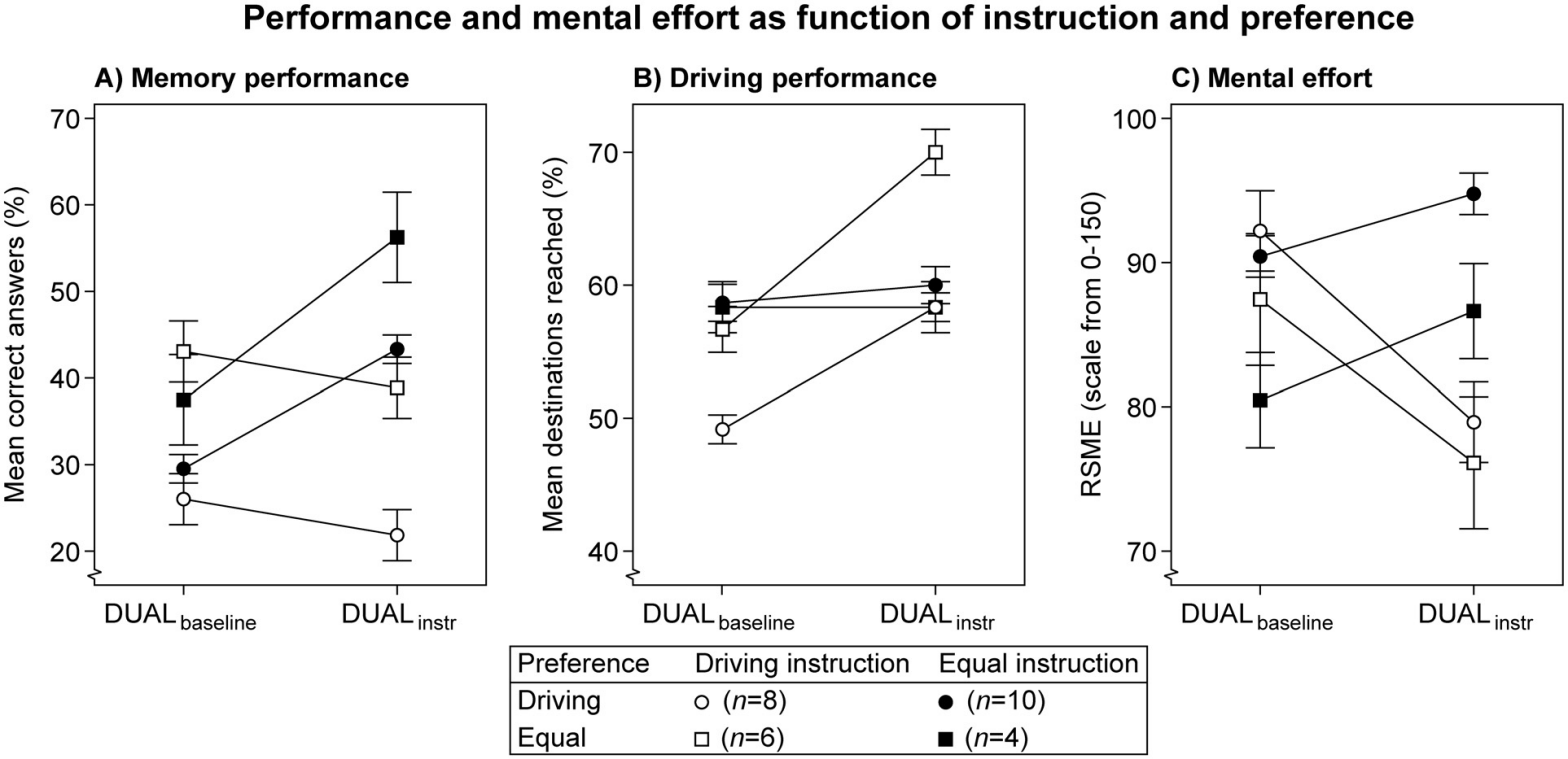
Results of the ‘driving’ and ‘equal’ priority instruction groups as function of preference. Lines are added for interpretation only. Error bars represent +/- 1 standard error of the mean, corrected for within-subject variability. The participant with a preference for the memory task in the 'free choice' group was omitted. Note: no instruction was provided in the DUAL_**baseline**_ condition.

The ‘driving’ and ‘equal’ instruction groups are compared to whether preferences influence how the instructions are followed. The ‘free choice’ group is omitted from this comparison, because conflicts with preferences are not applicable without an instruction. Fig 9 displays task performance and mental effort as function of Instruction and Preference.

As before, the priority instructions clearly caused different tradeoffs between performance and mental effort. The ‘equal’ instruction (closed symbols) shows increasing memory performance and stable driving performance, at the expense of increasing mental effort. By contrast, the ‘driving’ instruction (open symbols) shows slightly decreasing memory performance, increasing driving performance, and decreasing mental effort. A 2 (Instruction) × 2 (Preference) × 2 (Period) mixed ANOVA confirmed these observations, with a significant interaction between Instruction and Period on all measures (see Table 7).

**Table 7:** Summary of ANOVA results on performance and mental effort as function of instruction and preference.

	Memory performance			Driving performance			Mental effort		
Source	*F*(1,24)	*p*	*η*_p_^2^	*F*(1,24)	*p*	*η*_p_^2^	*F*(1,24)	*p*	*η*_p_^2^
Period	3.32	.081	.12	14.25	.001	.37	1.34	.26	.053
Instruction	2.51	.13	.095	.005	.95	< .001	.52	.48	.021
Preference	5.85	.024	.20	.42	.52	.017	1.10	.31	.044
Per × Instr	10.06	.004	.30	9.83	.004	.29	8.35	.008	.26
Per × Pref	.31	.59	.013	.077	.78	.003	.096	.76	.004
Instr × Pref	.30	.59	.012	.69	.41	.028	.18	.67	.008
Per × Instr × Pref	.13	.73	.005	.69	.42	.028	< .001	.99	< .001

Instr = Instruction, Per = Period, Pref = Preference.

Within each instruction group, all increments and decrements are in the same direction for both preferences (i.e., comparing circles vs. squares per instruction). As a result, no significant Preference × Period interactions were found, nor were there significant Instruction × Preference × Period interactions. The significant Instruction × Period interactions suggest that participants were able to follow the task priority instructions. Moreover, the absence of other significant interactions implies that task priority instructions were followed, regardless of preference.

Nonetheless, the magnitude with which the preferences separate within each instruction group (i.e., compare Figs 8 and 9) indicates that preferences did affect absolute performance and mental effort. In Fig 9A memory performance is higher with the 'equal' preference than with the 'driving' preference in both instruction groups. This was supported by a significant main effect of Preference on memory performance. No other main effects of Preference were found.

A significant effect of Period was found on driving performance. Fig 9B shows that the main driver for this effect is the 'driving' instruction group. Note, however, that the absolute performance level in all groups is still below that of the control group in Experiment 2 (see Fig 6B). If the task is fully learned, then the 'driving' instruction is expected to result in stable driving performance, whereas a decrement is expected with the 'equal' instruction (see Fig 7B). Therefore, the main effect of Period in the present experiment can be interpreted as a learning curve.

A closer inspection of the DUAL_baseline_ condition in Fig 9 indicates that the various groups differ in their baseline performance and mental effort. For example, in Fig 9B the group with a 'driving' instruction and a 'driving' preference shows lower driving performance in the DUAL_baseline_ condition than the other groups. This suggests that participants were not sufficiently trained to reach an equal performance level before being exposed to the dual-task conditions. We tested this observation by subjecting the DUAL_baseline_ data to a one-way ANOVA with four levels (i.e., the logical combinations of Instruction and Preference). In addition, a one-way ANOVA with four levels was conducted on DR_baseline_ and on the memory training data to examine single-task differences. No significant effects were found in either test. It seems that participants were not yet fully trained on the driving task before the DUAL_instr_ condition, but they were equally trained across the groups.

#### Resemblance between instructions and preferences

The previous section compared the ‘driving’ and ‘equal’ instruction groups to demonstrate that priority instructions were followed regardless of preferences. This section also includes the ‘free choice’ group, to investigate whether priority instructions resulted in task performance and mental effort comparable with the preferences on which the instructions were based. Within the ‘free choice’ group itself, participants with a ‘driving’ preference had lower memory performance and higher driving performance on the DUAL_baseline_ and DUAL_instr_ conditions. However, a 2 (Preference) × 2 (Period) ANOVA yielded no significant effects for both measures. Similarly, no significant effects were found on mental effort.

Two separate 2 (Instruction) × 2 (Period) mixed ANOVAs were conducted. One ANOVA concerned participants with a 'driving' preference within the 'driving' and 'free choice' instruction groups. The other ANOVA concerned participants with an 'equal' preference within the 'equal' and 'free choice' instruction groups. The results of these tests are summarized in Table 8. Participants with a 'driving' preference showed signicantly higher performance in the DUAL_instr_ condition (*M* = 61.25%, *SE* = 4.64) than in the DUAL_baseline_ condition (*M* = 56.25%, *SE* = 4.55). This effect reflects the learning curve on the driving task. Furthermore, a significant interaction between Instruction and Period was found on mental effort, again for participants with a 'driving' preference. The 'driving' instruction resulted in decreased mental effort from DUAL_baseline_ (*M* = 92.19, *SE* = 5.02) to DUAL_instr_ (*M* = 78.94, *SE* = 6.76), whereas the 'free choice' group showed increased mental effort from DUAL_baseline_ (*M* = 88.98, *SE* = 10.52) to DUAL_instr_ (*M* = 97.88, *SE* = 8.99). No other significant effects were found.

**Table 8:** Summary of ANOVA results on preferences with matching instructions.

	**Preference: 'driving'**								
	**Memory performance**			**Driving performance**			**Mental effort**		
**Source**	***F*(1,14)**	***p***	***η***_**p**_^**2**^	***F*(1,14)**	***p***	***η***_**p**_^**2**^	***F*(1,14)**	***p***	***η***_**p**_^**2**^
Period	1.17	.30	.077	4.88	.044	.26	.29	.60	.020
Instruction	2.16	.16	.13	1.22	.29	.080	.54	.48	.037
Per × Instr	.005	.94	< .001	3.47	.084	.20	7.55	.016	.35
	**Preference: 'equal'**								
	**Memory performance**			**Driving performance**			**Mental effort**		
**Source**	***F*(1,6)**	***p***	***η***_**p**_^**2**^	***F*(1,6)**	***p***	***η***_**p**_^**2**^	***F*(1,6)**	***p***	***η***_**p**_^**2**^
Period	2.01	.21	.25	2.03	.20	.25	.92	.37	.13
Instruction	.001	.97	< .001	1.91	.22	.24	.005	.95	.001
Per × Instr	1.99	.21	.25	.15	.71	.025	.72	.43	.11

Participants with an 'equal' instruction were excluded from the ANOVA on the 'driving' preference. Vice versa, participants with a 'driving' preference were excluded from the ANOVA on the 'equal' preference. Instr = Instruction, Per = Period.

To summarize, participants who acted according to their preference showed task performance similar to those with a matching instruction. The ‘driving’ instruction, however, resulted in decreased mental effort compared to the ‘free choice’ group. The latter group may have had doubts on how well they were expected to perform on the memory task. The presence of the ‘driving’ instruction may have resulted in more efficient use of energetic resources.

### Discussion

Experiment 3 has three main findings. Priority instructions have been followed, regardless of preference. Nonetheless, preference does influence memory performance, regardless of the instruction. Finally, the instructions have resulted in performance that resembles the preferences on which the instructions have been based. These findings lead back to the question why the ‘equal’ priority instruction was not followed in Experiment 2. We formulated two explanatory factors: conflicting preferences and lack of dual-task exposure. The successful manipulation of priority instructions in Experiment 3 appears to refute the factor of conflicting preferences.

However, through logical reasoning it must be concluded that both factors play a role. Suppose that preference has no effect on task performance. In that case, a lack of dual-task exposure would be the only explanation why participants in Experiment 2 have been unable to follow the priority instructions. However, the same amount of dual-task exposure has been given to participants in Experiment 1, yet they have been able to act according to their preference. This means preference must have played a role in Experiment 2.

Now suppose that preference is the only factor that has influenced following priority instructions in Experiment 2. In that case, an equally disruptive effect of preference would be expected in Experiment 3. Although the ‘equal’ preference has shown improved memory performance, also in the ‘driving’ instruction group, its influence has been too small to hinder the priority instructions. This means preference cannot be the only factor that influences following instructions. Together with the previous deduction, this suggests that the increased amount of dual-task exposure in Experiment 3 has decreased the effect of conflicting preferences on following priority instructions.

## General Discussion

The central question in this study was whether people differ in their preferences regarding task prioritization, and if so, whether these preferences influence the effectiveness of priority instructions. The results of three experiments show that people indeed have distinct preferences in an experimental dual-task setting (Experiment 1), which can be overruled by priority instructions, but only after a certain amount of dual-task exposure (Experiments 2 and 3).

**Fig 10:**
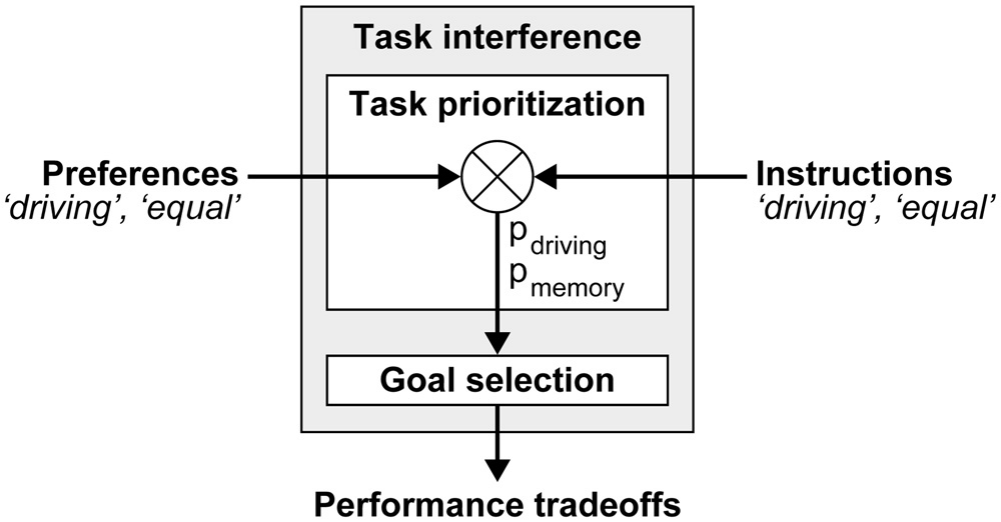
Model of task prioritization in the context of task interference.

Fig 10 provides an overview of the phenomena in this study. Combining two tasks with overlapping resources has created a situation of task interference. Performance tradeoffs are a direct consequence of task interference, in that task performance on one or both tasks is lower compared to single-task performance. A task prioritization process regulates which of the tasks suffers most from task interference, by setting priority levels for each task goal [7,8]. These priority levels in turn influence goal selection by the procedural resource, as illustrated previously in Fig 2. Experiment 1 demonstrates that preferences (i.c., ‘driving’, ‘equal’) influence the task prioritization process (i.e., the levels of p_driving_ and p_mem_), because these preferences have resulted in distinct performance tradeoffs. By contrast, the task prioritization processes in Experiments 2 and 3 have not only been a function of intrinsic preferences, but also of extrinsic instructions. We thus observed that the 'equal' instruction was not followed in Experiment 2, but it was followed in Experiment 3, after increased dual-task exposure. From this we speculated that if both preferences and instructions influence task prioritization, the relative weights of these factors on the priority levels should determine whether tasks are performed in favor of the instruction, or the preference. The next section summarizes how the weights of preferences and instructions on the relative task priority levels have differed between the experiments. This gives rise to an integration of TCT’s goal selection mechanism within a framework of regulatory control.

### Variable weight of preferences

**Fig 11:**
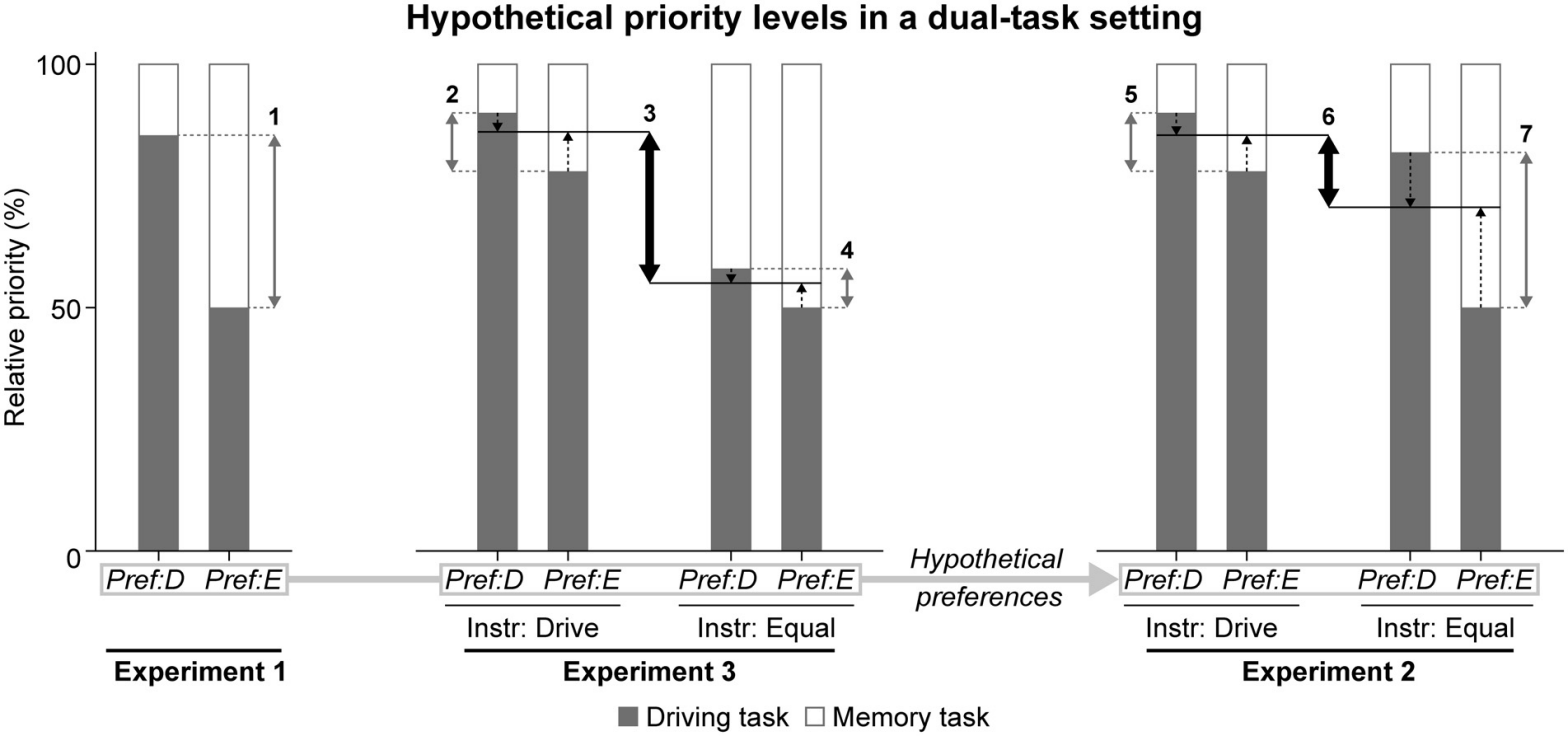
Hypothetical priority levels in three experiments. Pref:D and Pref:E correspond with ‘driving’ and ‘equal’ preferences, respectively. The preference distribution in Experiment 2 is based on Experiments 1 and 3. Arrows (1,2,4,5,7) correspond with differences in priority allocation as result of preference. Arrows (3,6) indicate differences as result of priority instruction, averaged over the number of preferences within each instruction. Dashed arrows point to the weighted average of preferences within an instruction. Numbered arrows are described in the text.

Preference appears to have affected the relative priority levels of each task goal (hereafter, ‘priority distribution’) with different weights throughout the experiments. Fig 11 shows an hypothetical priority distribution between the driving task and the memory task for each experiment, to illustrate our speculation on relative differences across the instructions and experiments.

In Experiment 1 preference has been responsible for distinct priority distributions (see arrow ‘1’). We interpret the ‘equal’ preference as a 50/50% distribution between the driving task (i.e., gray bars in Fig 11) and the memory task (i.e., white bars). The ‘driving’ preference cannot be represented as a 100/0% distribution, because memory performance scores above zero demonstrate that the memory task was still attended. Therefore, we interpret the ‘driving’ preference as an 80/20% priority distribution in favor of the driving task, corresponding with the priority levels p_2_ and p_1_ in the example of Fig 2.

An instruction should result in a similar priority distribution as the preference on which the instruction is based. In Experiment 3 differences in priority distribution have been caused by priority instructions (arrow ‘3’). Nonetheless, the higher memory performance with the ‘equal’ instruction demonstrates that preference did influence priority distribution (arrows ‘2’ and ‘4’). Therefore, it is safe to assume that preference has also played a role in Experiment 2 (arrows ‘5’ and ‘7’). In addition, the consistent distribution of preferences in Experiments 1 and 3 suggests that in Experiment 2, too, the majority of participants has had a ‘driving’ preference. These assumptions explain why the ‘driving’ instruction in Experiment 2 has resulted in a similar performance tradeoff as the ‘driving’ preference in Experiment 1. Moreover, if the majority of participants with an ‘equal’ instruction have acted according to their ‘driving’ preference, it becomes clear why task performance and mental effort did not deviate significantly from the ‘driving’ instruction (i.e., arrow ‘6’ is small compared to arrow ‘3’).

The variable weight of preferences may be explained by viewing priority distribution as the outcome of a judgment on task utility, which was a recurring theme in the verbal reports of Experiments 1 and 3. In general, people are known to only engage in behavior if the rewards associated with that behavior (e.g., enjoyment) outweigh the predicted energetical costs (e.g., mental effort) [30,36,37]. Accordingly, the predicted energetical costs will have outweighed the limited rewards in Experiment 2. However, in the second dual-task condition of Experiment 3 the energetical costs have likely been lower, due to increased task-switching efficiency [35]. Consequently, the evaluation of energetical costs and rewards has turned out favorably towards following the instructions in Experiment 3.

### Integrated model for task prioritization

Until now, the switching mechanism of TCT has assumed fixed goal priority levels (see Fig 1). If, however, preferences cause variability in priority distribution, and if preferences are the result of utility judgments, then the next question is how to link such judgments with TCT. Task performance has been related with cost-benefit mechanisms (i.e., utility judgments) in several theoretical accounts [16,30,32,33]. The Compensatory Control Model (CCM) [16,17], for example, describes the regulation of action in terms of a cost-benefit decision about the use of effort and the relative value of different goals. The higher one values a goal, the greater the willingness to spend additional effort on the corresponding task when its demands increase. An illustration of this cost-benefit decision is found in Experiment 2, where the driving task was protected against performance degradation, at the cost of decreased memory performance and increased mental effort.

**Fig 12:**
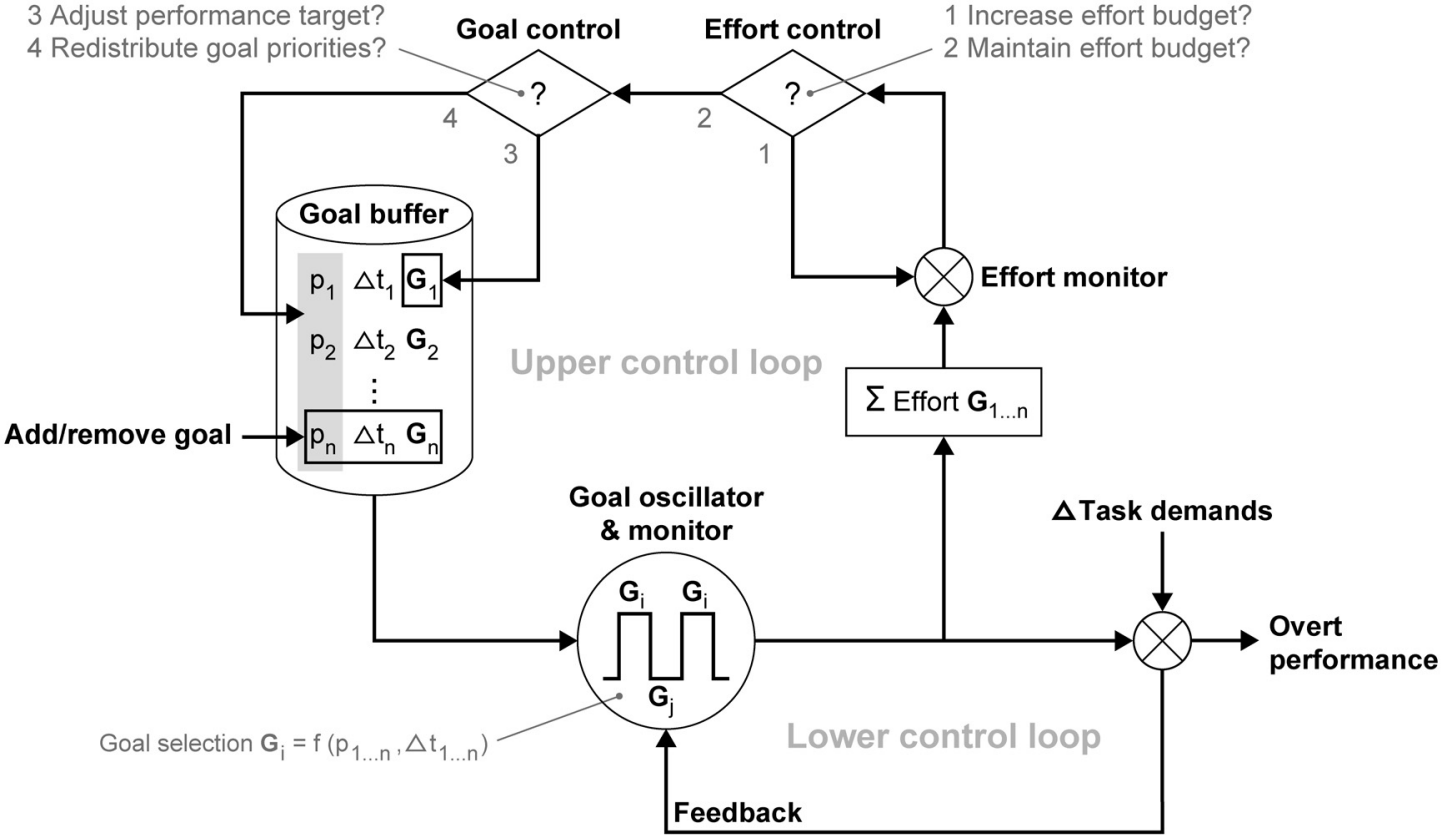
Integration of Threaded Cognition Theory [9] as goal oscillator within the Compensatory Control Model (adapted from [17] with permission).

We assume that cost-benefit decisions take place at a slower rate than the rapid switching mechanism described by TCT, analogous to the ‘slow’ and ‘fast’ systems of [38]. Contrary to other cognitive-energetic models, the CCM allows for an explicit temporal distinction by capturing the above regulatory process in two control loops. Fig 12 describes a preliminary integration of TCT within CCM. The upper control loop features a cost-benefit decision structure, which adjusts goal priority levels in the goal buffer. In the lower control loop, TCT is modeled as a goal oscillator that switches between goals, as prescribed by Fig 1. In line with CCM, the goal oscillator adapts its output by comparing overt performance with the selected goals from the goal buffer. The lower control loop ‘sees’ goal priority levels in the goal buffer as constants, even though they are occasionally adjusted by the upper control loop. Thus, the control loops in this integration operate in different time domains.

The adjustment of goal priority levels works as follows. An effort budget is used to compensate for sudden demand increments and resource decrements. The effort monitor compares the effort budget with the total effort level associated with the execution of all task goals. Therefore, the model in Fig 12 includes a summation of effort over iterations of partial task goal executions in the lower control loop. If the effort budget is insufficient to compensate for a discrepancy between intended performance and actual performance (e.g., failure to drive an intended route), CCM predicts a series of options [17]. The effort budget is either strategically raised to protect performance at the cost of fatigue (1), or lowered to prevent fatigue at the cost of task performance (2). These strategies are found with the 'equal' and 'driving' instructions in Experiment 3, respectively. Task performance decrements are either effectuated by adjusting the performance target of the current goal (3), or by displacing the current goal with a competing goal. We interpret goal displacement as a redistribution of priority levels (4). Ideally, priority instructions have a large effect on priority (re)distribution. Deviations from this ideal distribution are found when preferences result in an alternative cost/benefit decision.

### Implications & future research

This explorative study provides several starting points for future research. From a theoretical perspective, a validation is needed of the proposed integration of TCT within CCM. We acknowledge that the proposed integration is currently not detailed enough to be implemented in the cognitive architecture in which TCT is modeled. However, recent studies show promising attempts at predicting single-task effort [39,40], which provide an opportunity to test how effort drives task prioritization in concurrent multi-tasking. Specifically, these attempts may address the summation of effort in Fig 12, which features a transition from a fast process (e.g., goal oscillator, TCT) to a slow process (e.g., effort and goal control).

From a methodological perspective, the consequence of asking people afterwards about their preference, is that this procedure may result in unequal sample sizes, and low numbers in certain conditions. We acknowledge that this occurred in the present study. Indeed, when viewed per experiment, a low n may have reduced the reliability of the observed patterns. Looking across the experiments, however, we have observed several consistent patterns, yielding confidence in our overall results. For example, the distributions of the 'driving' and 'equal' preferences were consistent across Experiments 1 and 3. This helped to interpret the results of Experiment 2.

The question remains how to prevent unequal samples sizes when inquiring individual preferences. Asking people about their preferences beforehand is not a straightforward solution, because it may bias performance later on. Therefore, participant selection in future research benefits from having an inconspicuous method to predict preferences. If such method would exist, then knowledge on the likely distribution of preferences may prove instrumental in determining how many prospective participants should be recruited.

The causal role of preference on task prioritization was established through logical deduction from the combined results of Experiments 1 through 3. However, this deduction does not exclude other interpretations, such as the possiblity that participants assess their own performance, and then base their preference report on that. This issue, too, may be resolved by a method to predict preferences.

This study questioned the widespread assumption that people follow priority instructions in a dual-task setting. The assumption appears to be correct, provided that enough dual-task exposure is provided beforehand. A practical question, then, is exactly how much dual-task exposure is required before a conflicting priority instruction ‘wins’ against preference, and to what extent this is task- and context-dependent. In the traffic context, optimal safety requires drivers to prioritize the driving task at all times. This premise is not feasible for police officers, due to the dominant role of radio communication [1,41]. Although Dutch police officers do receive special driving training, they have to learn in the field how to balance between driving and listening. The present study suggests that these officers benefit from dual-task training to meet the implicit ‘equal’ priority instruction of police work, especially if this instruction conflicts with their task prioritization preferences.

## Supporting Information

S1 Fig Rating Scale Mental Effort in English and in Dutch. As presented onscreen. Adapted from [34] for computer use.
(TIF)

S2 Fig Hockey's Compensatory Control Model. Reprinted from [17] with permission.
(PNG)

S1 File Datasheet for Experiments 1, 2 and 3. (XLSX)
