# Supplementary figures and images for: Task Prioritization in Dual-Tasking: Instructions versus Preferences
Source: PLoS One. 2016 Jul 8;11(7):e0158511. doi: 10.1371/journal.pone.0158511 (PMC4938591; doi:10.1371/journal.pone.0158511)

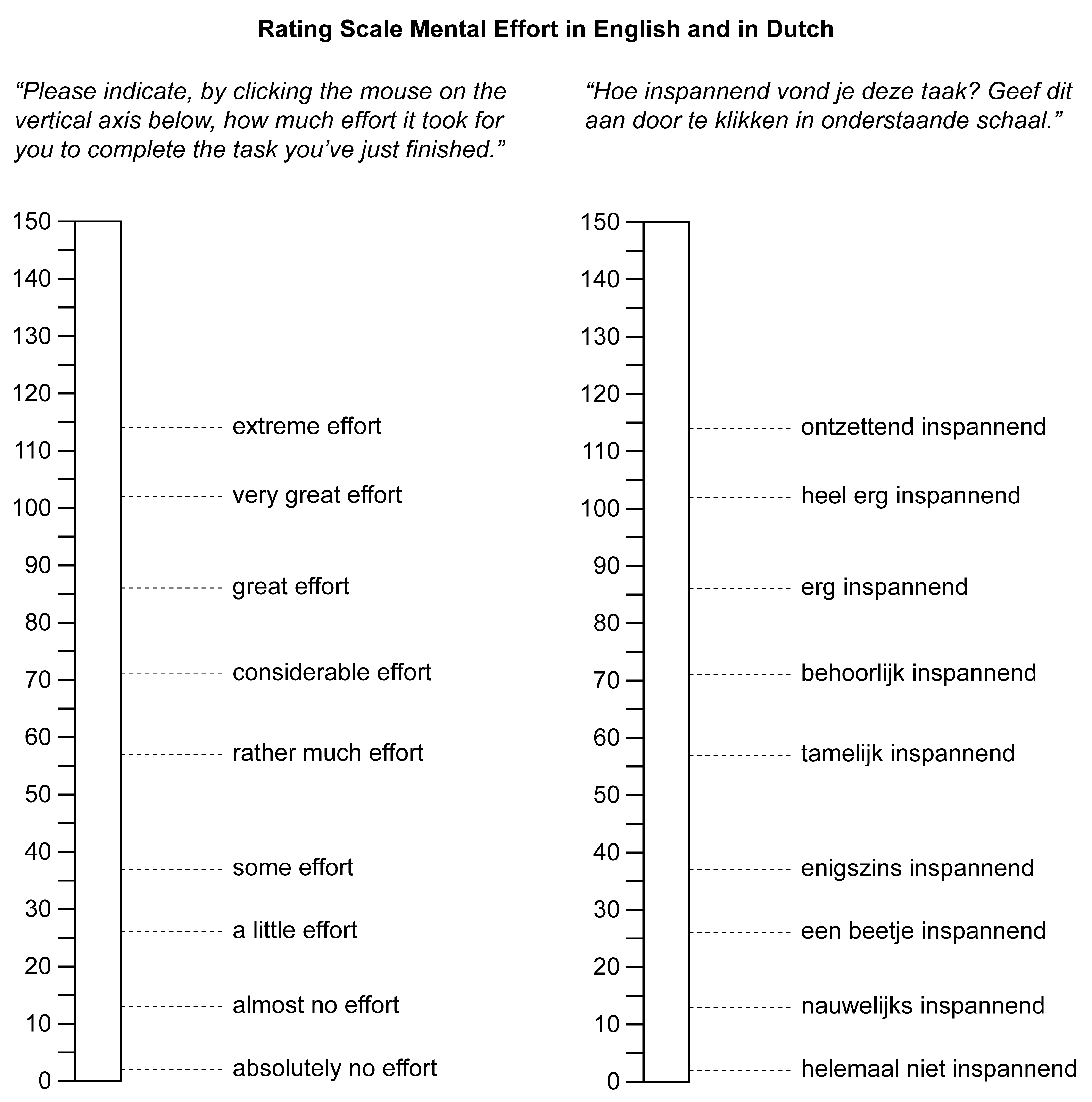

Supplement: S1 Fig — As presented onscreen. Adapted from [34] for computer use. (TIF) [file pone.0158511.s001.tif]

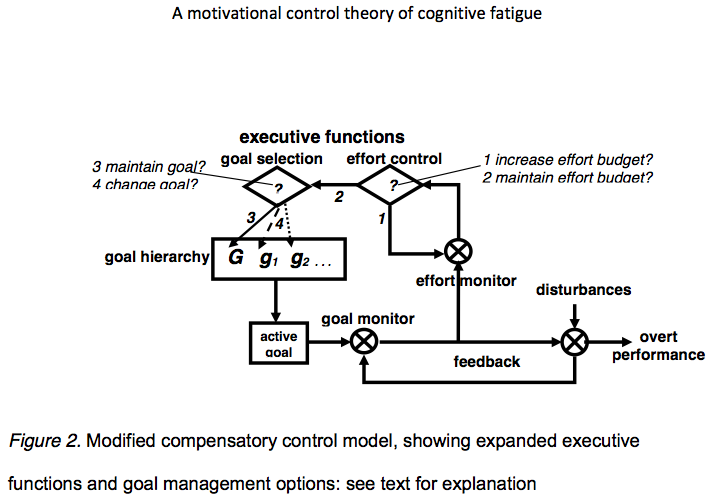

Supplement: S2 Fig — Reprinted from [17] with permission. (PNG) [file pone.0158511.s002.png]
